# Biodegradable Metal-Based Stents: Advances, Challenges, and Prospects

**DOI:** 10.3390/jfb16090315

**Published:** 2025-08-29

**Authors:** Lifeng Sun, Yuanyuan Zeng, Zhengyu Shen, Chongsheng Yue, Yahan Yang, Jia Gao, Junhao Zhang, Qi Yuan, Limei Cha

**Affiliations:** 1 Biotechnology and Food Engineering Department, Guangdong Technion-Israel Institute of Technology, Shantou 515063, China; sun07453@gtiit.edu.cn (L.S.); zeng08550@gtiit.edu.cn (Y.Z.); yang24624@gtiit.edu.cn (Y.Y.); 2 Chemical Engineering Department, Guangdong Technion-Israel Institute of Technology, Shantou 515063, China; shen09616@gtiit.edu.cn (Z.S.); gao24091@gtiit.edu.cn (J.G.); 3 Materials Science and Engineering Department, Guangdong Technion-Israel Institute of Technology, Shantou 515063, China; yue09947@gtiit.edu.cn (C.Y.); junhao.zhang@gtiit.edu.cn (J.Z.); 4 Materials Science and Engineering Department, Southern University of Science and Technology, Shenzhen 518055, China

**Keywords:** metal stents, biodegradation, magnesium alloys, iron alloys, zinc alloys, biocompatibility, metabolism

## Abstract

Cardiovascular disease is a leading cause of global mortality. Percutaneous coronary intervention, which involves the placement of stents to restore blood flow in narrowed arteries, is a widely used treatment. However, traditional stents, such as bare metal stents and drug-eluting stents, can lead to long-term complications such as restenosis, inflammation, and thrombosis. Biodegradable metallic vascular stents, with their superior mechanical properties, excellent biocompatibility, and gradual degradation in vivo, hold significant potential for the treatment of coronary artery disease. This review provides a comprehensive overview of the current research status and challenges. Firstly, it outlines the design principles and performance evaluation methods for biodegradable stents, which focus on mechanical properties, chemical characteristics, corrosion behavior, and biocompatibility. Furthermore, it summarizes the material features, degradation mechanisms, and metabolic behavior of three primary biodegradable metals—magnesium alloys, iron alloys, and zinc alloys—and discusses critical issues such as the degradation rate of different alloys and the development of zinc alloys. Finally, based on the current achievements and challenges of studies on biodegradable metal-based stents, this article proposes some optimization strategies and research prospects.

## 1. Introduction

Cardiovascular disease (CVD) is responsible for approximately 17.9 million deaths annually [1], with coronary artery disease being particularly prevalent. Common methods used to treat arterial occlusions include balloon angioplasty, percutaneous coronary intervention (PCI), and coronary artery bypass grafting. Among these, PCI is one of the most rapid and effective techniques. This procedure involves placing a vascular stent at the site of arterial narrowing to expand the artery, alleviate obstructions, and restore blood flow. The use of coronary stents has grown significantly, with over 90% of PCI procedures involving coronary stents [2,3], and approximately two million coronary artery stent implantation surgeries performed globally each year [4]. Since the 1980s, metallic-based vascular stents have made significant progress as a key treatment for coronary artery disease. The development of vascular stents has progressed through three generations: the first generation is bare-metal stents (BMSs), the second generation is drug-eluting stents (DESs), and the third generation is biodegradable vascular stents (BVSs). Bare-metal stents effectively reduced the risk of acute occlusion, but their long-term use revealed issues such as restenosis and in-stent thrombosis. Drug-eluting stents, which are coated with antiproliferative drugs, can reduce the incidence of restenosis; however, the long-term presence of permanent stents within blood vessels leads to numerous complications, including inflammatory responses, in-stent restenosis, and late-stage thrombosis. To address these challenges, biodegradable vascular stents have garnered increasing attention.

From a clinical perspective, an ideal biodegradable stent should possess the following characteristics: provide sufficient mechanical support in the early stages to prevent vascular occlusion; gradually degrade after fulfilling its mechanical support function, ultimately being safely absorbed by the body; avoid long-term complications and late-stage lumen loss; and restore the natural physiological function of blood vessels, such as maintaining normal blood flow and supply.

To achieve these goals, it is essential to thoroughly investigate the degradation process of stents and understand the synergistic effects between various degradation factors. Therefore, this study aims to (1) provide an overview of the basic knowledge framework for this research area, which can help beginners with different backgrounds to gain a comprehensive understanding of this field and find intersection research points, and (2) summarize the advancements and emerging frontiers within this interdisciplinary field, identifying cross disciplinary intersections and their corresponding subbranches, thereby facilitating more in depth and effective research and innovation. In particular, the research methods, details, and key points we report are not only guided by improving material properties but also by the practical and safety requirements of biodegradable stents. Focusing on the degradable properties of stents, this review provides a detailed summary of the research methods and recent advancements in biodegradable metallic vascular stents from the perspectives of chemical engineering, biology, and materials science. Key aspects include factors influencing in vitro dynamic corrosion experiments, biocompatibility testing methods, the features of typical biodegradable alloys, and the challenges encountered in practical applications, as well as the structural design of stents. Based on the major achievements and unresolved issues in current research, potential future research directions and technological breakthroughs are proposed.

## 2. Properties and Related Research Methods for Degradable Metal Stents

The design of degradable metallic cardiovascular stents primarily considers four aspects: mechanical properties of materials (strength, hardness, elongation, elastic modulus, etc.), chemical characteristics (biodegradability, ion release, absorbability, pH value, etc.), biological properties (biocompatibility, hemocompatibility, etc.), and structural design of stents (cell design, connection design, etc.). All these factors will be discussed in the following Section 2, Section 3, Section 4 and Section 5.

Section 2.1 focuses on the mechanical properties of materials/stents and standard methods used to test these properties. Section 2.2 describes in vitro simulation experiments, including the typical method of placing the stent in a simulated cardiovascular environment to investigate its corrosion rate and metabolic byproducts. By monitoring parameters such as mechanical properties and stent degradation, the feasibility and potential clinical applications of the stent can be preliminarily evaluated. A significant amount of research has been conducted on these properties over the past two decades; the tables in Appendix A summarize the test results for alloy materials used in the manufacturing of biodegradable stents.

### 2.1. Mechanical Properties and Testing Parameters

The mechanical performance of a stent is mainly tested based on the following aspects: material analysis and testing (composition analysis, tensile and ductility mechanical properties, corrosion resistance, etc.), stent intrinsic performance (radial recoil performance, longitudinal shortening performance, expansion uniformity, radial support performance, flexibility, fatigue resistance), and integrated performance of the stent with the delivery system. Each test focuses on a certain property of the material/stent.

Table 1, which is based mainly on Ref. [5] and has been cited several times [6,7], outlines the performance requirements of materials used for biodegradable vascular stents. The strength, ductility, and corrosion resistance of vascular stent materials are typically characterized by ultimate tensile strength (UTS), yield strength (YS), elongation (EL), and corrosion rate. Some studies [8,9,10,11,12,13,14] provide the standards and methods for testing these mechanical behaviors.

Besides the mechanical criteria for raw materials, additional mechanical properties of the stents need to be tested, including (1) radial recoil performance, (2) longitudinal shortening performance, (3) radial support performance, and (4) flexibility of vascular stents. The definitions of these terms, their roles in stents, and some critical parameters are briefly described below.

**Figure 1:**
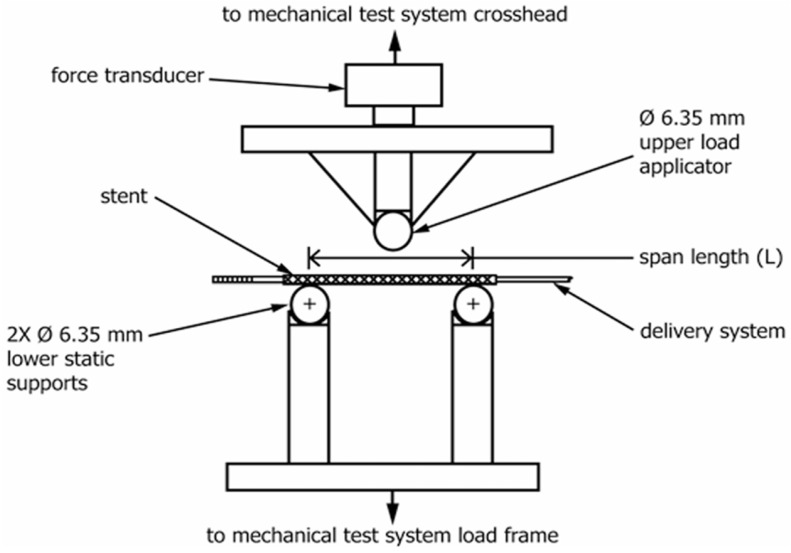
Schematic diagram of testing flexibility [12]. Reprinted with permission Ref. [12]. Copyright 2014 ASTM International.

(1) Radial recoil performance, also known as intrinsic elastic recoil, refers to the change in the diameter of the vascular stent from balloon expansion to balloon deflation. It is usually represented by the radial recoil rate. Stents with a lower radial recoil rate can reduce vascular wall injury and the occurrence of restenosis.
(2) Longitudinal shortage performance is usually represented by the longitudinal shortage rate, which refers to the change in length of the expansion of the vascular stent from the non-deployed state to the nominal diameter. In general, the clinically acceptable longitudinal shortage rate for vascular stents is 0~20%.
(3) Radial support performance refers to the ability of vascular stents to resist vascular wall constriction. It is represented by the corresponding compression load when the compression reaches 50% of the unloaded stent’s outer diameter [16].
(4) Flexibility refers to the longitudinal bending ability. Bending flexibility of a deployed stent may be one measure of its ability to flex with a vessel or to conform to the natural curvature of a vessel. The specimen is loaded onto a three-point bend fixture. The specimen is supported from below by two static supports separated by a known span and bent by a force applied at the top and midway between the lower supports, as shown in Figure 1. The bending flexibility of the stent is obtained from force-versus-deflection plots and/or midspan bending moment versus midspan curvature plots.

The uniformity of expansion is also an important parameter of a stent. It refers to the difference between the largest and smallest diameter measurements on a single stent. Uniformity of expansion measurements should be assessed at three longitudinal locations (mid-length and close to each end) at each of two circumferential orientations with a separation of about 90°. The uniformity of expansion reflects both the deviation from circularity of the cross-section and unintended differences in diameter along the stent length.

When the stent delivery system is inflated to the maximum recommended inflation pressure, the diameters of both the proximal and distal ends of the balloon are larger than that of the rest of the stent. This is known as the ‘Dogbone Effect’ or ‘Dogboning’ or Edge effect [17]. Clinically, such a phenomenon is not desirable because the two ends of the inflated balloon can damage the vessels. Therefore, during the expansion process, the deformation of the stent edges should be as consistent as possible with the rest of the stent.

The fatigue resistance test evaluates the ability of the stent structure to maintain integrity under severe physiological loads. The generation of internal stress in vascular stents stems from the implantation of stents in curved arteries, radial expansion, arterial contraction, and hemodynamic shear forces caused by blood flow. Fatigue resistance aims to demonstrate that the implant will not fail in the in vivo environment it encounters during its service life, ensuring the dimensional and structural integrity of the implant during its service life. The fatigue resistance of stents depends mainly on the fatigue performance of the stent material, but the fatigue performance of the stent is also affected by the stent’s structural design.

### 2.2. Methods and Parameters of the In Vitro Dynamic Immersion Test

To simulate the corrosion behavior of biodegradable metal materials under physiological conditions, researchers have developed numerous in vitro corrosion testing systems. For example, one enables precise modification of the physical and chemical surroundings in the system, which closely replicates the human physiological environment. Additionally, it can be used to manipulate hemodynamic parameters to comprehensively evaluate the performance of the stent in vitro [18].

Given the complexity of physiological environments, in vitro experiments must consider several key factors, including the type of test solutions for immersion experiments, the solution’s volume-to-surface area ratio (V/S), hemodynamic conditions, pH levels, dissolved oxygen concentration, temperature, and immersion duration. These parameters should be tailored to the specific experimental context to minimize discrepancies between in vivo and in vitro results. Such adjustments are essential for providing accurate insights into the performance and degradation behavior of metal-based stents [19].

#### 2.2.1. Selection of Immersion Solution for the Corrosion Test

The composition of the immersion solution is of paramount importance. Inorganic ions and organic components in the solution play a crucial role in the degradation behavior of metals and alloys. Variations in the concentrations of inorganic salts in simulated physiological environments can significantly influence the corrosion behavior and corrosion rate of materials. For instance, during the degradation of zinc alloys, the presence of hydrogen, carbonate, phosphate, and chloride ions affects the stability of corrosion products such as Zn(OH)_2_ and ZnO, thereby altering the composition of the corrosion layers [20].

This ion-dependent degradation mechanism is not unique to zinc. Similarly, during the corrosion of magnesium alloys, the penetration of Cl^−^ ions disrupts the protective layer composed of Mg(OH)_2_ and MgHPO_4_, rendering it unstable and leading to the formation of soluble MgCl_2_. This process accelerates both the progression of corrosion and the overall corrosion rate [21,22,23,24,25]. Table 2 shows some important constituents of the extracellular fluid for researchers to refer to when preparing the immersion solution.

Additionally, to ensure the fidelity of the simulation and the reliability of the data, the composition and concentration of test solutions must be carefully optimized to replicate in vivo conditions before proceeding with experiments. Various solution systems are employed to simulate body fluids depending on the specific test objectives. Commonly used solutions include Hank’s, SBF, HBSS, m-SBF, SBP, HEPE, DMEM, PBS, and media for in vitro organ culture [27,28,29,30,31,32,33,34,35]. Some of their specific test objectives and compositions are shown in Table 3.

#### 2.2.2. Ratio of Solution Volume to Sample Surface Area (V/S)

Once an appropriate solution composition is selected, the volume in which the sample is immersed relative to its surface area becomes a key consideration for maintaining solution stability. The ASTM G31-72 standard recommends that the ratio of solution volume to the surface area of the sample be between 20 mL/cm^2^ and 40 mL/cm^2^ [36]. This ratio is essential for replicating an environment that reflects the oxidation behavior of the sample, avoiding oxygen depletion and the accumulation of corrosion products [33]. By adhering to these standards, researchers can obtain more reliable and consistent experimental results that are not skewed by solution exhaustion effects.

#### 2.2.3. Hemodynamic Parameters

**Figure 2:**
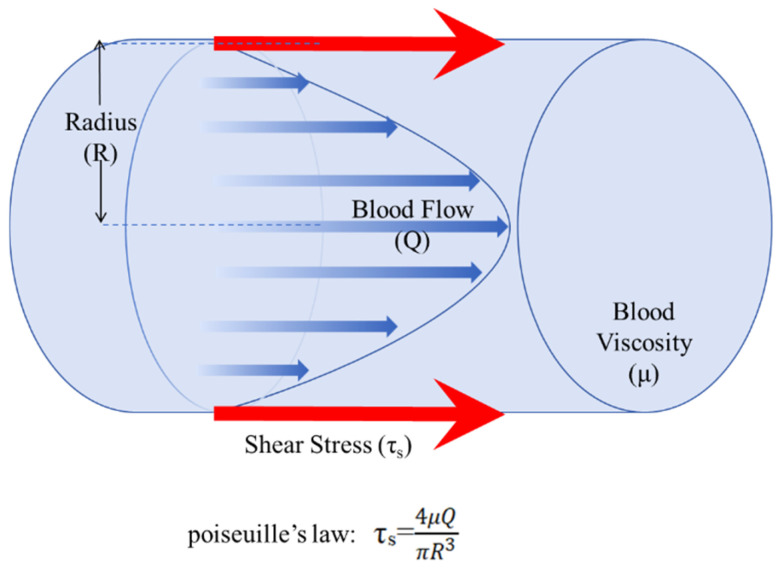
Cross-sectional schematic diagram of a blood vessel illustrating hemodynamic shear stress (τ_s_, shown as red arrows), the frictional force per unit area acting on the inner vessel wall and on the luminal surface of the endothelium as a result of the flow of viscous blood (Q, shown as blue arrows) Based on data from Ref. [26].

**Figure 3:**
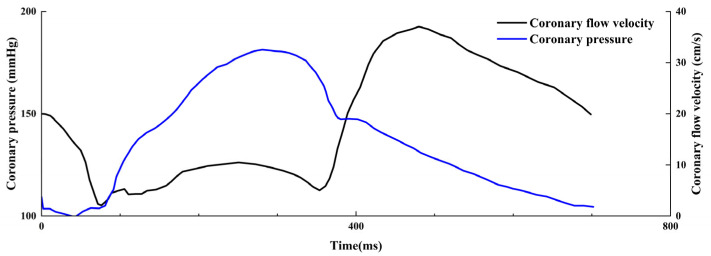
Relationship between intra-coronary pressure and flow velocity [38]. Based on data from Ref. [38]. Only an approximate relationship is shown.

Beyond static solution properties, simulating the dynamic flow environment encountered by implants such as stents in the bloodstream is crucial for a comprehensive assessment. In the initial stage of stent implantation, the stent’s exposure to flowing blood results in blood erosion on the stent surface predominating over electrochemical corrosion [33]. In this situation, the corrosion rate and mechanism of the stent vary with changes in applied shear stress. As shear stress increases, the overall corrosion rate accelerates [37]. Vascular shear stress caused by blood flow can be calculated using Poiseuille’s law [37], as shown in the Figure 2. The commonly used ranges of hemodynamic parameters in simulated experiments are presented in Table 4, corresponding to the vascular shear stress calculation. The approximate relationship between intra-coronary pressure and flow velocity is also given in Figure 3, which helps researchers construct in vitro simulations. 

**Figure 4:**
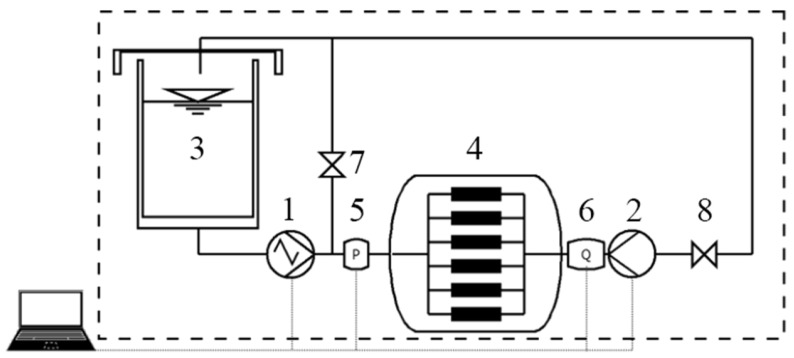
Schematic design of the bioreactor (1: pulsatile pump; 2: static counter pressure pump; 3: reservoir; 4: stented arteries in a culture chamber; 5: pressure transducer; 6: flow rate transducer; 7: bypass valve; 8: counter pressure valve.) [40]. Adapted with permission from Ref. [40]. Copyright 2015 Elsevier.

To accurately replicate these dynamic conditions in vitro, Figure 4 shows a system that can be used to control hemodynamic parameters for evaluating in vitro samples [39,40]. It employs two pumps; the main pump generates a pulsating flow by adjusting its rotating speed, while the second pump operates at a constant speed to maintain internal pressure levels and regulate backflow. This setup enables adjustment of the flow rate, velocity curve, arterial pressure, and resistance, ensuring that the hemodynamic conditions closely mimic those of the human body [26].

#### 2.2.4. The Effect of pH, Dissolved Oxygen Concentration, and Temperature

In addition to replicating fluid flow, precise control of the fundamental chemical and physical parameters of the simulated body fluid is vital for mimicking the physiological milieu and obtaining relevant corrosion data. These critical parameters include pH, dissolved oxygen (DO) concentration, and temperature.

##### The Effect of pH on In Vitro Experiments

The pH of blood and extracellular fluid is a key parameter in the human body. The normal physiological pH range is narrow; the typical pH values are presented in Table 5 [41]. In in vitro experiments, pH plays an important role in the corrosion rate of metal surfaces. During metal corrosion, a high pH value leads to the formation of a compact corrosion layer on the surface, thereby reducing the corrosion rate [42]. For example, H_2_ and OH^−^ are produced during the degradation of magnesium in a medium. If the pH in the solution is not controlled, the local pH around the magnesium surface will continue to increase. When the local pH exceeds 11, a magnesium oxide layer forms on the surface, creating a barrier between the metal and the corroding solution, slowing down the degradation process [43,44]. In contrast, at a pH of approximately 7–10, acidification resulting from the anodic dissolution of Zn can disrupt the previously formed passivation layer, and the cathodic reaction rate decreases, slowing down the overall corrosion rate of zinc [44].

Therefore, the concentrations of H^+^ and OH^−^ in the solution have a substantial impact on corrosion dynamics. Maintaining the pH of simulated body fluid within the physiological range during immersion experiments ensures a more accurate evaluation of metal corrosion behavior, thereby improving the reliability of experimental results.

##### The Effect of Dissolved Oxygen (DO) Concentration on In Vitro Experiments

Parallel in importance to pH is the role of dissolved oxygen, as the corrosion of stents is significantly influenced by the concentration of dissolved oxygen (DO), which affects the oxidation process and the composition of corrosion products. The DO concentration in the human body is presented in Table 5. Thomas et al. [45] showed that dissolved oxygen plays a crucial role in the primary cathodic oxygen reduction reaction, altering the corrosion potential and, consequently, the corrosion rate [46]. In particular, the corrosion behavior of zinc strongly depends on the DO concentration.

Similarly, oxygen availability critically shapes the corrosion pathways of other metals. For instance, the corrosion rate of iron is closely linked to local DO levels. During oxygen reduction, Fe^2+^ ions react with OH^−^ to form insoluble hydroxide precipitates. Under alkaline and oxygen-rich conditions, simulating body fluids, Fe^2+^ can be further oxidized to Fe^3+^, leading to the formation of Fe(OH)_3_. In the presence of chloride ions, Fe(OH)_3_ undergoes hydrolysis, precipitating as Goethite (α-FeO(OH)), while Fe(OH)_2_ reacts with FeO(OH) to form Fe_3_O_4_ [47].

The specific composition of corrosion products significantly impacts the assessment of material corrosiveness, as different products may have varying effects on cellular metabolism [48]. To improve the accuracy of in vitro studies, the DO concentration in simulated body fluids used for immersion experiments is carefully controlled, ensuring a more accurate representation of in vivo conditions. This ensures a more accurate evaluation of stent corrosion behavior and the potential effects of corrosion products on cellular metabolism, thereby enhancing the reliability of experimental results.

##### The Effect of Temperature on In Vitro Experiments

Temperature is another critical factor influencing experimental outcomes, as it directly affects reaction rates during the degradation process. The normal human body temperature is approximately 37 °C, and excessively high experimental temperatures can accelerate corrosion, compromising the accuracy of corrosion rate assessments. For instance, an increase of just 3 °C (from 37 °C to 40 °C) can lead to a roughly 50% increase in the corrosion rate of high-purity magnesium [42]. Additionally, temperature may influence protein adsorption, which in turn affects subsequent biological reactions [49].

Considering their combined influence, physiological parameters such as pH, dissolved oxygen concentration, and temperature can be categorized into first-order and second-order correlations. First-order correlations, including temperature and pH, have a direct impact on the degradation rate, while second-order correlations, such as dissolved oxygen concentration, influence the formation of the corrosion layer and the composition of corrosion products [26].

**Table 5:** Acid–base, body temperature, and oxygen partial pressure of the extracellular fluid. Based on data from Ref. [50].

	Normal Value	Normal Range	Approximate Short-Term Nonlethal Limit	Unit
Acid–base	7.4	7.3–7.5	6.9–8.0	pH
Body temperature	98.4 (37.0)	98–98.8 (37.0)	65–110 (18.3–43.3)	°F (°C)
Oxygen partial pressure	40	35–45	10–1000	mmHg

**Figure 5:**
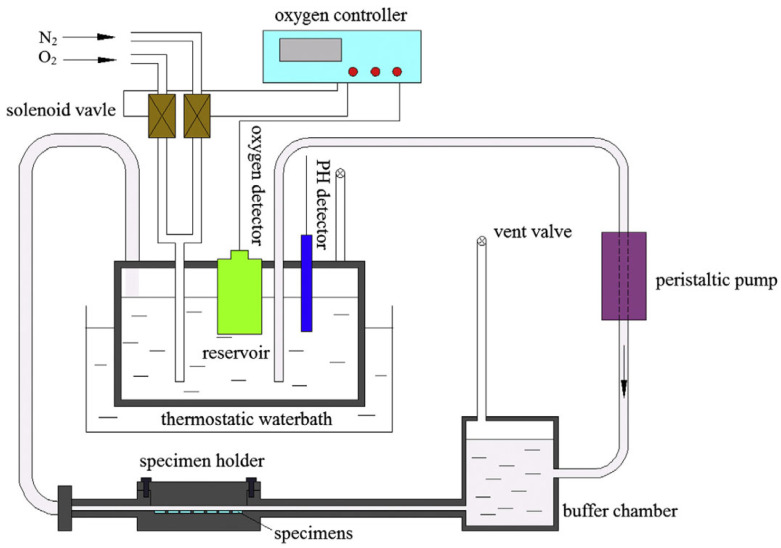
Diagram of dynamic corrosion test devices [51]. Reprinted with permission from Ref. [51]. Copyright 2011 Elsevier.

Based on the above description of pH, DO and temperature, a dynamic corrosion test device diagram can be designed, as shown in Figure 5.

To accurately simulate the physical and chemical environment of the human body, the system employs precise control mechanisms. Temperature is monitored using a detector integrated into the oxygen sensor and regulated by a constant-temperature water bath. Dissolved oxygen concentration is adjusted by introducing oxygen and nitrogen, which are chemically inert in the corrosion reaction, ensuring that the oxygen level remains within physiological conditions. Meanwhile, a buffer solution stabilizes the pH, preventing significant fluctuations during the experiment. The pH of the simulated body fluid is continuously monitored with a pH meter and adjusted as needed by adding diluted sodium hydroxide or hydrochloric acid solutions [52]. This integrated approach to environmental control ensures a controlled experimental environment, improving the reliability and relevance of corrosion assessments.

## 3. Research Progress on Degradable Materials

The first bare metal stent used for expanding the coronary artery was a permanent stent made of 316L stainless steel [53]. Subsequently, cobalt–chromium and platinum–chromium alloys largely replaced stainless steel due to their superior strength, allowing for thinner struts while maintaining adequate radial strength. This design enhancement effectively increases the stent’s inner cavity area and enhances radiopacity for better visibility during implantation. Moreover, cobalt–chromium alloys exhibit minimal metal ion release, reducing the risk of allergic reactions in patients [54]. However, long-term presence of permanent stents can lead to persistent inflammation, intravascular restenosis, and stent thrombosis. To address these issues, biodegradable vascular stents, which are made of either polymer or metal, have garnered attention. Polymer biodegradable stents exhibit lower mechanical strengths compared to metal stents of the same size. Thicker struts may achieve equivalent radial support while reducing the effective internal cavity area. The Absorb series of polymer biodegradable stents, for example, showed poorer clinical endpoint data compared to widely used Xience Co-Cr stents and was withdrawn from the market in 2017 [55]. In the realm of biodegradable metals, three types have been selected after stringent screening for biodegradability, biocompatibility, and metal stability: iron, magnesium, and zinc alloys [14]. A thorough understanding of the compositions of magnesium, iron, and zinc alloys, as well as their degradation and metabolism mechanisms in the human body, is crucial for controlling degradation rates and processes effectively, as well as achieving optimal biocompatibility and enhancing biosafety. This section outlines the three alloy systems, providing an overview of material characteristics, in vitro corrosion mechanisms, and in vivo metabolism mechanisms. Since the performance of a stent is determined not only by the properties of the material itself but also by its structural design, different materials often require tailored design strategies. For instance, magnesium alloy stents with relatively weak radial support can be made thicker, while iron alloy stents with slow degradation rates tend to minimize total mass and increase contact area. Through synergistic optimization of material and structure, the final stent products can reach comparable overall performance. Therefore, in the summary of material development, the focus is primarily on the differences in material properties and their respective improvement strategies.

### 3.1. Degradable Magnesium Alloys

#### 3.1.1. Introduction to the Magnesium Alloys

Magnesium alloys utilized in degradable vascular stents primarily include the AE series (containing aluminum and rare earth elements), the AZ series (containing Aluminum and zinc), the Mg-RE series (enriched with rare earth elements), and the Mg-Li series [56]. The diverse mechanical properties of these alloys—yield strength (YS), ultimate tensile strength (UTS), elongation (EL), and hardness (HV)—are summarized in Table 6.

The AE21 alloy from the AE series, which contains 2% aluminum and 1% rare earth elements, was one of the earliest materials employed in biodegradable magnesium stents. Despite its pioneering status, AE21 degrades rapidly, increasing the risk of early recoil and restenosis post-stenting compared to 316L stainless steel alloys. Research efforts are directed towards moderating the degradation rate to address these challenges [57].

The AZ series includes alloys such as AZ31B, which contains 3% aluminum and 1% zinc and undergoes deformation treatment, and AZ91, with 1% zinc and a higher aluminum content of 9%. The addition of aluminum and zinc not only boosts the strength and corrosion resistance of these alloys but also leads to the formation of the β-phase (Mg17Al12). With increasing aluminum content, the β-phase becomes more prevalent, and a continuous β-phase enhances the corrosion resistance [58]. Simultaneously, the presence of trace zinc in the magnesium matrix effectively promotes non-basal slip (slip occurring on non-basal planes), lowers the stacking fault energy, and thereby improves the plastic deformation capability of the material. However, the aluminum content raises concerns, as its acceptability in the human body is limited to about 1 g per person per year [59], which might restrict its broader clinical application.

This limitation has spurred the development of the Mg-RE series, such as the WE43 (Mg-Y-Gd-Nd alloy) first used clinically by Biotronik [60,61,62,63,64,65,66], and ZE21B (Mg-Zn-Y-Nd alloy, post-deformation) developed by Zhengzhou University [67], along with JDBM (Mg-Nd-Zn-Zr alloy, extrusion-treated) developed by Shanghai Jiao Tong University [51,68]. In WE43, the addition of yttrium and neodymium not only improves corrosion resistance but also avoids cytotoxicity. The Magmaris stent, originally known as DREAMS 2G, made from WE43, is extensively studied and shows promising flexibility in clinical applications. ZE21B, with lower zinc and yttrium contents, exhibits a higher corrosion resistance compared to WE43, resulting in the corrosion potential of cast ZE21B alloy (−1.76 V) being higher than that of cast WE43 alloy (−1.95 V). Its properties can be further enhanced by various processing techniques and heat treatments, such as the sub-rapid solidification process, cyclic extrusion compression (CEC) [69], hot extrusion [70], and the HTHE (long-time high-temperature heat treatment, large reduction specific heat extrusion) process [71]. JDBM benefits from added neodymium, which reduces stacking fault energy and provides a pinning effect on the slip systems of the alloy matrix, while zirconium acts as a grain refiner, improving the toughness and biocompatibility of the alloy. Additionally, JDBM’s surface is covered with a dense protective layer (Mg12Nd phase), which significantly enhances corrosion resistance and helps maintain uniformity in the corrosion process during initial immersion.

Emerging Mg-Li series alloys, such as the Mg-Li-Zn ternary alloy [72] and the Mg-Li-Al-Zn quaternary alloy [73], are being explored primarily for their mechanical properties and corrosion resistance, achieved through solution strengthening, grain refining, and dispersion strengthening. Nonetheless, their toxicity and biocompatibility are still under investigation and require further confirmation.

Despite the improvements in mechanical properties and corrosion resistance brought by these alloying elements, magnesium alloy stents still do not meet all clinical demands due to their rapid degradation rate in vivo, significant reduction in radial force after implantation, and the tendency for the implantation site to retract post-implantation [74]. In addition to adjusting the chemical composition, other techniques are also used to modify corrosion resistance. For example, coating technologies used to improve corrosion resistance and biocompatibility can be divided into three major categories: Chemical Conversion coatings, Micro-Arc Oxidation (MAO) coatings [75], and Electrophoretic Deposition coatings. Chemical Conversion involves using chemicals such as phosphate [76], magnesium hydroxide [77,78], magnesium fluoride (MgF_2_) [79], and silane-based compounds [25] to modify the surface of the alloy and form protective layers. External polymer coatings often occur as a functional top layer in composite coating structures (e.g., MAO/PLGA/paclitaxel), which involve materials like polylactic acid (PA) [80], polylactic acid–glycolic acid [81], polycaprolactone [82], polytrimethylcarbonate [83], and polyurethane [84,85]. Among these, micro-arc oxidation forms a ceramic-like oxide layer with improved mechanical and corrosion properties; however, the outer porous layer compromises long-term corrosion resistance, rendering it insufficient as a standalone coating for implantable devices [75]. Magnesium fluoride coatings, on the other hand, significantly reduce the corrosion rate (e.g., from 0.874 mm/y to 0.094 mm/y) and have shown excellent in vivo biocompatibility without triggering adverse immune responses [77,78]. Silane-based coatings, such as those using bis[triethoxysilyl]ethane (BTSE) and γ-aminopropyltriethoxysilane (APS), have demonstrated the ability to reduce platelet adhesion, improve tissue compatibility, and promote endothelialization [25]. In terms of polymer coatings, phytic acid, a naturally derived organic coating, provides favorable endothelial cell adhesion and low hemolysis rates, although its inherent corrosion resistance is relatively limited [80]. Finally, polylactic acid (PLA) and its copolymer PLGA not only serve as protective barriers but also act as drug carriers. For instance, PLGA-loaded paclitaxel coatings in AM-2.1 and PLLA-sirolimus systems effectively extend degradation time and control neointimal hyperplasia [81]. These coating strategies are critical in enhancing the corrosion resistance and biocompatibility of magnesium alloy stents.

#### 3.1.2. Mechanism of Mg Corrosion In Vitro

Magnesium and its alloys degrade in aqueous environments, undergoing the following electrochemical reactions [24]:

Mg ↔ Mg^2+^ + 2e^−^ (anodic partial reaction); (1)

2H_2_O + 2e^−^ ↔ H_2_ + 2OH− (cathodic partial reaction); (2)

Mg + 2H_2_O ↔ Mg_2+_ + H_2_ + 2OH− (overall reaction); (3)

Mg + 2OH^−^ ↔ Mg(OH)_2_ (product formation). (4)

Dissolved metal ions react with phosphoric acid in an aqueous solution, leading to the following reaction:

Mg^2+^ + HPO_4_^2^− → MgHPO4 (5)

**Figure 6:**
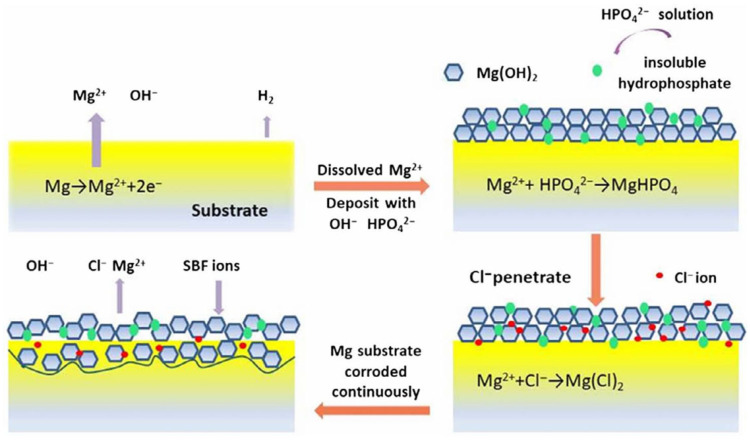
A schematic illustration of corrosion mechanisms in Mg-based alloys in a simulated body fluid (SBF) solution [25]. Adapted with permission from Ref. [25]. Copyright 2019 Wiley.

Mg^2+^ reacts with OH^−^ to form slightly soluble Mg(OH)_2_, which is deposited on the substrate surface [24]. As corrosion progresses, a protective film, primarily consisting of Mg(OH)_2_ and MgHPO_4_, is formed on the material’s surface, as depicted in Figure 6 [25]. The formation of the film has a positive effect on the corrosion performance [86].

Dissolved metal ions and ionized calcium (Ca^2+^) ions can also react with other anions in the solution to form carbonates, phosphates, chlorides, and other corrosion products [20,45,87]. This reduces the corrosion rate [20].

Mg(OH)^2^ + 2Cl^−^ ↔ MgCl^2+^ 2OH^−^ (6)

During corrosion, the hydroxide film gradually becomes unstable, thereby facilitating the penetration of Cl^−^ ions within this quasi-protective film [21,22,23]. Therefore, Mg(OH)_2_ is converted to a soluble substance, such as MgCl_2_, by the reaction (6) [24]. As the transformation progresses, corrosion resistance declines [25]. 

#### 3.1.3. Mechanism of Magnesium Metabolism in the Human Body

Magnesium plays a critical role in the cardiovascular system [88]. In general, magnesium homeostasis in the human body is regulated through a dynamic balance of intestinal absorption, exchange with bone, and renal excretion. Intracellular magnesium forms a vital complex with ATP, enabling its involvement in numerous biological processes, including protein synthesis, cell replication, and energy metabolism [89].

Magnesium is chemically active and readily oxidized in vivo, with its reaction pathways mainly aligning with the in vitro corrosion mechanisms detailed in Section 3.1.2. A notable characteristic of magnesium implants is their ability to react with body fluids, producing magnesium hydroxide and hydrogen gas [90]. However, rapid degradation and excessive hydrogen release may lead to tissue damage [91].

Additionally, factors such as dissolved oxygen, proteins, amino acids, chloride ions, and hydroxide ions influence degradation mechanisms, including magnesium salt formation and transformation. During degradation, amino acids, proteins, and lipids adsorb onto magnesium implant surfaces, altering the degradation rate. At later stages, magnesium implants gradually dissolve in body fluids, releasing magnesium ions that are metabolized for critical processes such as bone formation, protein synthesis, and DNA synthesis. Excess ions and corrosion byproducts are excreted via the urinary system [92].

Normal serum magnesium levels range from 0.74 to 1.07 mmol/L. Symptoms of hypermagnesemia, such as neuromuscular disturbances, typically manifest when serum magnesium concentrations exceed 2 mmol/L [93]. In a clinical trial evaluating the magnesium alloy stent WE43, serum magnesium levels in subjects peaked at 1.7 mmol/L on the second day post-implantation and returned to normal within 48 h [94].

In vivo studies show that magnesium alloy stents initially form Mg(OH)_2_ layers, followed by replacement of Mg^2+^ with calcium ions, resulting in the formation of amorphous calcium–phosphorus complexes. Through metabolic processes, magnesium is converted into well-tolerated products such as chlorides, oxides, sulfides, and phosphates, with complete degradation occurring months after implantation [14]. Nonetheless, the long-term (over one year) degradation behavior of magnesium stents and the physiological effects of their products remain poorly understood [95].

Research on the in vivo corrosion behavior of magnesium alloys remains limited [96], with no consensus on factors such as grain size, secondary phase composition, and distribution. While chemical, immersion, and implantation experiments provide insights, their findings are sometimes contradictory, emphasizing the need for targeted analysis in specific scenarios [97].

### 3.2. Degradable Iron Alloys

#### 3.2.1. Introduction to the Iron Alloys

Compared with magnesium alloys (refer to Table 6 in Section 3.1.1), the iron alloy for manufacturing degradable vascular stents offers significant mechanical advantages. Additionally, iron has been demonstrated to have good biocompatibility for vascular applications for up to 18 months [98]. Iron stents can be safely implanted without significant obstruction of the stented vessel caused by inflammation, neointimal proliferation, or thrombotic events, as demonstrated by in vivo animal experiments [98,99,100,101].

**Figure 7:**
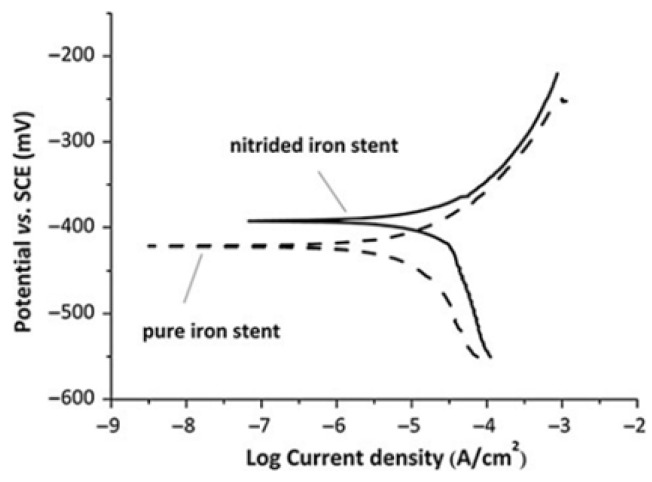
Potentiodynamic polarization curves of the nitrided iron stent and the pure iron stent [102].

Currently, the most thoroughly investigated alloy in this system is the ferrinitrided material (second-phase particles ${Fe}_{4}N$ scattered in the pure iron matrix). As evidenced by data in Table 7 [102], using the universal testing machine (CMT6502), TC700-101 radial strength testing machine (Machine Solution Inc., Flagstaff, AZ, USA), and HXD-1000TMC digital microhardness tester, the tensile strength, radial strength, and stiffness of ferrinitrided materials surpass those of pure iron, and significantly exceed those of magnesium alloys. Consequently, ferrinitrided stents provide enhanced support and occupy a smaller volume compared to stents of equivalent mass made from magnesium alloys and pure iron [103]. Moreover, ferrinitrided stents also exhibit considerable benefits in terms of in vitro electrochemical corrosion rates. Figure 7 [102] displays the dynamic electrochemical polarization curve recorded by a three-electrode system in a Gamry PCI4/300 electrochemical workstation. The detailed parameters are summarized in Table 8 [102]. The corrosion rate of ferrinitrided iron is almost double that of pure iron due to micro-galvanic corrosion facilitated by the interaction between the matrix and the finely dispersed particles of ferric nitride compounds within the diffusion layer, which markedly accelerates stent degradation [104]. Specifically, the IBS stent, composed of iron nitriding and developed by Lifetech Scientific (Shenzhen, China) Co., Ltd. using vacuum plasma nitriding technology, has been clinically validated through experiments involving the implantation of the stent in the iliac arteries of piglets [102].

However, a primary concern with such ferrinitrided stents is their slow degradation rate. After endothelialization and vascular remodeling—the adaptive process through which blood vessels respond to hemodynamic changes through cellular recombination and extracellular matrix remodeling [104]—the remaining stents may become focal points for late thrombosis and chronic inflammation [105,106,107]. Furthermore, the use of ferrinitrided stents may interfere with magnetic resonance imaging (MRI) processes and leave residues due to uneven degradation, complicating subsequent medical interventions [108,109]. Some studies propose enhancing the properties of the iron matrix using alloying techniques such as the Fe-Mn-Pd alloy, which incorporates dispersed intermetallic phases into the iron matrix to improve its mechanical strength and accelerate corrosion [110,111,112]. Table 9 [113] outlines the mechanical properties of the intermetallic reinforced iron matrix ($\varepsilon_{u}$, uniform elongation; $\varepsilon_{f}$, elongation at fracture; sht, solution heat treated for 2 h at 1100 °C in an argon atmosphere followed by water quenching; ht1, aged for 1 h at 500 °C to achieve a microstructure of tempered martensite, retained austenite, and Pd-IMP precipitates; ht2, briefly annealed in the c-phase region for 10 min at 700 °C and subsequently aged for 10 h at 500 °C to promote IMP formation in a high austenitic phase state). The iron matrix after intermetallic reinforcement exhibits even better mechanical properties than ferrinitrided material. However, the addition of new metallic elements, particularly Mn, might reduce the material’s cytocompatibility [51,114,115]. Additionally, while low concentrations of Mn in Fe-Mn alloys have shown promising mechanical properties and biocompatibility, no significant corrosion was noted nine months post-implantation [116]. However, comprehensive data from animal studies and clinical trials to substantiate the viability of such materials is currently lacking.

#### 3.2.2. Mechanism of Fe Corrosion in Vitro

The degradation of ferric nitride alloys in vivo involves the following electrochemical reactions:

Anodic reaction: Fe → Fe^2+^ + 2e^−^, (7)

Cathodic reaction: O_2_ + 2H_2_O + 4e^−^ → 4OH^−^, (8)

The iron ions produced by corrosion diffuse to the material’s surface, while oxygen and phosphate ions in the solution diffuse from the surrounding environment to the surface. Both Fe_2_O_3_ and Fe_3_O_4_ are located in the core of the nitrided iron platform, whereas Fe_3_(PO_4_)_2_ is found only in the outer layer. A portion of the iron ions diffuses into the surrounding tissues primarily as α-FeOOH and γ-FeOOH phases [87].

Fe^2+^ + 2OH^−^ ↔ Fe(OH)_2_, (9)

(10) $$3\mathrm{Fe}(\mathrm{OH}{)}_{2}+\frac{1}{2}O_{2}⟶{\mathrm{Fe}}_{3}O_{4}+3H_{2}O\text{,}$$

(11) $$2\mathrm{Fe}(\mathrm{OH}{)}_{2}+\frac{1}{2}O_{2}+H_{2}O⟶2\mathrm{Fe}{\left(\mathrm{OH}\right)}_{3}\text{,}$$

Fe(OH)_3_ ⟶ FeOOH + H_2_O, (12)

2Fe(OH)_3_ ⟶ Fe_2_O_3_ + 3H_2_O, (13)

3Fe^2+^ + 2HPO_4_^2−^ ⟶ Fe_3_(PO_4_)_2_ + 2H^+^, (14)

In the human body, the corrosion products participate in metabolism, undergo the Fenton reaction, and generate free radicals [117,118,119,120]. Under aerobic conditions, Fe^2+^ reacts with oxygen to form a superoxide anion, which subsequently forms H_2_O_2_. The interaction of H_2_O_2_ with the hydroxyl groups of Fe^2+^ products results in further chemical changes.

Fe^2+^ + O_2_ → Fe^3+^ + O_2_^⦁^^−^ (15)

Fe^2+^ + O_2_^ ⦁^^−^ + 2H^+^ → Fe^3+^ + H_2_O_2_, (16)

Fe^2+^ + H_2_O_2_ → Fe^3+^ + OH^⦁^ + OH^−^, (17)

#### 3.2.3. Mechanism of Iron Metabolism in the Human Body

Iron is one of the essential trace elements in the human body. It is predominantly distributed in red blood cells and the liver, where it is stored in intracellular protein complexes such as ferritin and hemosiderin. In addition to dietary iron absorbed in the intestine, macrophages play a critical role in recycling iron released from senescent or damaged cells, a process primarily managed in the spleen before storage in the liver. Notably, the majority of circulating iron originates from the bone marrow–red blood cell–macrophage recycling loop, with only minimal contributions from dietary intake. Iron excretion from the body is exceedingly limited, primarily occurring via the gastrointestinal tract. This rate remains stable regardless of systemic iron levels. Additionally, trace amounts of iron may be lost through sweat, urine, bile, and menstrual blood [121].

The normal serum iron concentration in healthy individuals ranges from 10 to 30 µmol/L [122]. Given the small size and mass of iron-based stents and their slow degradation rate, the amount of iron released during degradation is significantly lower than the systemic iron load (~447 mg/L), mitigating the risk of systemic iron toxicity. The iron stent system has relatively low toxicity and a slow degradation rate. This conclusion is further supported by several animal studies on iron-based stents, which demonstrate favorable biocompatibility and minimal adverse systemic effects [108].

Similar to the in vitro corrosion processes described in Section 3.2.2, major in vivo iron corrosion products include Fe_3_O_4_, Fe_2_O_3_, FeOOH, Fe(OH)_3_, and Fe_3_(PO_4_)_2_. These products are sparingly soluble at physiological pH (~7.4), but a dynamic solubility equilibrium allows trace levels of dissolved iron ions to be removed via systemic circulation, enabling continued dissolution of the insoluble products. However, the complete dissolution of iron oxides and hydroxides may take years, resulting in slow degradation of iron-based stents through solubility equilibrium [108].

Beyond solubility equilibrium, macrophage activity may also contribute to the in vivo degradation of iron stents. Macrophages, which are abundant in the body, possess the ability to migrate through tissues, blood vessels, and lymphatic vessels, engulfing foreign materials and pathogens for digestion. Studies suggest that macrophages can internalize insoluble iron corrosion product particles surrounding the stents and further interact with them. A proposed pathway involves macrophages engulfing iron particles, processing them into hemosiderin, and migrating from the stent site to the vascular adventitia before entering nearby lymph nodes via lymphatic vessels [100]. Another hypothesis posits that macrophages transport the processed iron to the liver, where excess iron is excreted into bile in the form of ferritin or heme via lysosomal exocytosis [123].

Despite these insights, the entire metabolic pathway of iron and its corrosion products remains inadequately elucidated, with existing information predominantly derived from isolated experimental observations and speculative models. Comprehensive studies are needed to elucidate the entire degradation and metabolic process.

### 3.3. Degradable Zinc Alloys

#### 3.3.1. Introduction to the Zinc Alloys

Compared with magnesium and ferrinitrided materials, the development of degradable zinc alloys for vascular stents is still in its early stages. The degradation rate of zinc alloy stents lies between that of iron and magnesium alloys, offering an intermediate rate more closely aligned with the natural vascular recovery cycle. Zinc is highly biocompatible, with the recommended daily intake ranging from 2–3 mg/day for infants to 8–11 mg/day for adults [124,125]. According to Vojtech et al. [126], the daily quantity of zinc released from the degradation of Zn alloy stents is approximately 1.5 mg, well below the toxicity threshold of 100–150 mg/day. Moreover, zinc ions can promote vascular endothelial growth, thereby aiding the healing and regeneration of vessels post-stent implantation [127], potentially offering benefits similar to those of magnesium alloys but to a greater extent.

**Figure 8:**
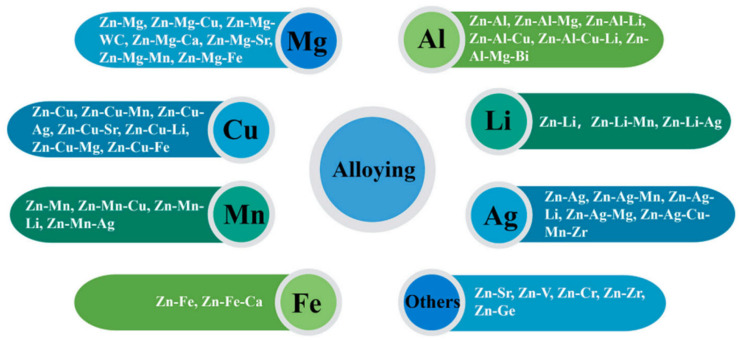
Common alloying elements of Zn alloys mainly include Mg, Al, Cu, Li, Mn, Ag, Fe, Sr, V, Cr, Zr, and Ge. They can form binary alloys, ternary alloys, quaternary alloys, and even quinary alloys with Zn [128]. Reprinted with permission from Ref. [128]. Copyright 2024 Elsevier.

Zinc alloys exhibit significant enhancements in mechanical properties through grain refinement achieved via alloying. The alloy systems under consideration include Zn-Mg, Zn-Al, Zn-Cu, Zn-Mn, Zn-Ag, Zn-Fe, and Zn-Li series, as illustrated in Figure 8 [128]. These alloys have also shown progress in improving fatigue resistance, aging resistance, corrosion resistance, antibacterial properties, and hydrophilicity, while maintaining low cytotoxicity [128]. Table 10 provides a summary of various mechanical properties (yield strength, ultimate tensile strength, elongation, and hardness) of materials suitable for vascular stents within these zinc alloy systems. Notably, Zn-Mg, Zn-Al, Zn-Li, and Zn-Cu alloys show considerable improvements in tensile mechanical properties, especially those containing Li and Al, which demonstrate the most pronounced strengthening effects [122]. Regarding degradation rates, the inclusion of Mg, Al, Cu, and Li achieves desirable rates (degradation rate ≈ 20 μm/year), whereas those with Mn, Ag, and Fe degrade too slowly [128].

Despite these advantages, zinc alloys have limitations due to their relatively low mechanical properties, which prevent them from withstanding the dynamic pressures within cardiac vessels. In particular, in high-stress environments such as vascular stenosis, the inherent weakness of zinc alloys may lead to deformation or failure of the stent, thereby compromising its long-term stability and effectiveness. Furthermore, the limited ductility of zinc alloys restricts their applicability in complex vascular structures. Compared to the other two alloys discussed before, the potential for mechanical property enhancement through elemental doping in zinc alloys is restricted, possibly due to inefficient grain refinement in as-cast zinc alloys, generally characterized by large grain size and suboptimal microstructural qualities [128]. To address these issues, studies recommend refining grain size, controlling the distribution and size of second-phase particles, and optimizing crystal orientation through advanced manufacturing processes. Techniques such as pressure infiltration [143], spark plasma sintering [144], additive manufacturing [145,146,147], electro-deposition combined with heat treatment [148], equal channel angular extrusion (ECAP) [148,149,150], static pressure extrusion (HE) [143], and high-pressure torsion [150] have been applied to enhance the material properties of zinc alloys.

#### 3.3.2. Mechanism of Zn Corrosion in Vitro

In an aqueous solution, pure zinc undergoes the following electrochemical reactions:

Zn + OH^−^ = Zn(OH)_ads_.+ e^−^, (18)

Zn(OH)_ads_.+ 2OH^−^ = Zn(OH)_3_^−^ + e(rds), (19)

Zn(OH)_3_^−^ + OH^−^ = Zn(OH)_4_^2−^, (20)

Zn(OH)_4_^2−^ = Zn(OH)_2_ + 2OH^−^, (21)

Zn + 2OH^−^ = Zn(OH)_2_ + 2e^−^, (22)

Zn + 2OH^−^ = ZnO + H_2_O + 2e^−^, (23)

According to the corrosion reaction, solid corrosion products such as Zn(OH)_2_ and ZnO are formed. The stability of these compounds is influenced by the concentration of hydrogen, bicarbonate, phosphate, and chloride ions in the corrosive environment [20]. Additionally, various ions in the environment can react with zinc, forming other Zn-containing compounds [151].

Recent studies on Zn alloys have shown that after in vitro degradation tests, elements such as Zn, P, O, C, Cl, and Ca are detected in the corrosion products, indicating the presence of carbonates [88,127,152]. In a simulated humoral environment containing carbonate, zinc undergoes the following reaction [87]:

ZnO + 2H^+^ = Zn^2+^ + H_2_O, (24)

Zn^2+^ + H_2_CO_3_ = ZnCO_3(s)_ + 2H^+^, (25)

5Zn^2+^ + 2HCO^3−^ + 6H_2_O = Zn_5_(OH)_6_(CO_3_)_2(s)_ + 8H^+^, (26)

2H_2_ + 2HCO^3−^ + H_2_O + 5ZnO_(s)_ = Zn_5_(OH)_6_(CO_3_)_2(s)_, (27)

The formation of an insoluble film on the surface can significantly hinder the dissolution of zinc, thereby influencing its corrosion behavior [149]. In the corrosive environment, chloride (Cl^−^) and phosphate (P) ions present in the corrosion products may result from ionic interactions between various acidic ionic species, such as chloride ions and phosphate groups [153].

In a Cl^−^-rich simulated body fluid environment, the primary corrosion product formed is zinc hydroxychloride (Zn_5_(OH)_8_Cl_2_·H_2_O), also known as simonkolleite. The formation of simonkolleite enhances corrosion resistance, as it creates a protective layer on the zinc surface. This process occurs through the following reaction [44]:

5ZnO + 2Cl^−^ + 6H_2_O = Zn_5_(OH)_8_Cl_2_H_2_O + 2OH^−^·ZnO + 2Cl^−^ + 6H_2_O = Zn_5_(OH)_8_Cl_2_·2H_2_O + 2OH^−^

In a simulated phosphate-containing fluid environment, the HPO_4_^2−^ ion forms an insoluble compound, ZnHPO_4_, thereby inhibiting further dissolution of zinc [152].

#### 3.3.3. Mechanism of Zinc Metabolism in the Human Body

Zinc is widely distributed across various cell types in the human body, where it serves essential catalytic, structural, and regulatory functions. The absorption and excretion of zinc in the gastrointestinal tract play a significant role in maintaining zinc homeostasis in the human body. Zinc absorption occurs primarily in the small intestine via a carrier-mediated mechanism. However, determining the exact proportion of absorbed zinc is challenging because significant amounts of zinc are also secreted back into the intestine during this process [154]. Zinc excreted via the gastrointestinal tract constitutes approximately half of the total zinc excretion. While substantial amounts of zinc are secreted through bile and intestinal secretions, most are subsequently reabsorbed. Other pathways for zinc excretion include urinary output, as well as losses through exfoliated skin and hair. These processes might contribute to the slow removal of additional zinc.

Zinc is chemically active and readily oxidized within the body. Zinc alloy implants degrade rapidly, but inert corrosion byproducts formed during the degradation process may accumulate on the surface of the alloy, inhibiting further degradation and thereby slowing the degradation rate. At physiological pH, the oxidation of Zn primarily results in the formation of Zn(OH)_2_. ZnO is generated as the local pH increases beyond 8.3 [134]. Additionally, bicarbonate ions (HCO_3_^−^) in body fluids react with Zn^2+^ to produce ZnCO_3_. Corrosion products of pure Zn in vivo mainly include ZnO, ZnCO_3_, and Zn_3_(PO_4_)_2_.

Studies have demonstrated that pure zinc can degrade safely and steadily in the body over extended periods. However, the precise mechanisms underlying the degradation of Zn and zinc-based stents, as well as the metabolic pathways of the resulting byproducts, require further investigation [155,156].

## 4. Biosafety of Metal-Based Stents

To ensure the safety of implantable medical devices, researchers typically conduct a series of assessments, including biocompatibility testing, animal experiments, and clinical trials. Biocompatibility is defined as the ability of a material to perform its intended function within the host without inducing mechanical injury, toxic reactions to surrounding tissues, or immune rejection [157].

For vascular stents, biocompatibility is usually evaluated through both in vitro and in vivo testing, focusing on the material’s interactions with biological fluids and cells. Particular attention is given to its effects on blood and its components, including hemolysis, cytotoxicity, and activation of immune responses [158].

### 4.1. Hemocompatibility

Hemocompatibility is one of the most critical criteria for the success of biomaterials used in vivo when they come into contact with blood. It involves not only the degree of platelet adhesion and activation but also the hemolysis ratio, which assesses the extent of red blood cell rupture or damage caused by interaction with the medical material. According to the ISO 10993-4 [159] standard, this ratio must be less than 5% to ensure the safety of the material [160,161]. Hemolysis is typically influenced by various factors, including the chemical composition, pH levels, and metal ion concentrations of the material [162]. Platelet adhesion is assessed by measuring the number of platelets adhering to the material’s surface. Excessive platelet adhesion may indicate a thrombogenic tendency, as it promotes platelet activation, which in turn facilitates thrombus formation [163]. In general, lower hemolysis ratios and fewer adhered platelets indicate superior hemocompatibility.

Since stents are implanted in the cardiovascular system, it is vital to determine whether they exert any adverse effects on the blood. Poor hemocompatibility of stents can lead to several complications. For instance, as blood flows over the stent’s surface, it may trigger red blood cell rupture or plasma protein adsorption, leading to platelet activation. Activated platelets can further initiate the intrinsic coagulation pathway, resulting in thrombin generation and subsequent clot formation [164]. Furthermore, poor hemocompatibility of implants may trigger adverse reactions such as complement activation and leukocyte damage, potentially leading to thrombosis and hemolysis. Thus, hemocompatibility testing is a fundamental experiment for defining the biocompatibility of biomaterials [162].

Some studies define biocompatibility based solely on cytotoxicity results, without evaluating hemolysis, platelet adhesion, or coagulation. Such an approach is insufficient for a comprehensive assessment of material biocompatibility. In practice, human or animal blood, often sourced from volunteers, is typically used for testing to simulate physiological environments and verify the biocompatibility of the material [165]. These tests not only help evaluate the material’s performance during blood exposure but are also a fundamental prerequisite for determining its suitability for clinical applications. Table 11 summarizes the hemolytic testing results of stents made from iron, zinc, magnesium, and some of their alloys.

As shown in Table 11, iron and zinc materials exhibit good to excellent hemocompatibility in hemolysis tests conducted for vascular stents made of iron, zinc, magnesium, and their alloys. The hemolysis rates for Fe and Fe-X binary alloys remain below 5%, demonstrating excellent anti-hemolytic properties and strong hemocompatibility of iron-based stents. In particular, Fe30Mn6Si and Fe30Mn alloys exhibit hemolysis rates below 2%, qualifying them as non-hemolytic according to ASTM F756-08 [175] standards. Zinc and its alloys, including Zn–0.8Cu, Zn–0.8Mn, and Zn–0.8Li, also show hemolysis rates well below 5%. Alloying with Cu, Mn, or Li effectively reduces the hemolysis rate of zinc. Notably, the Zn–1X binary alloy containing Mg, Ca, or Sr achieves exceptionally low hemolysis rates (<0.2%), reflecting outstanding hemocompatibility [176]. In contrast, magnesium materials show a more complex performance profile. Pure magnesium and certain magnesium alloys, such as Mg–1Zn–1Mn, exhibit significantly high hemolysis rates, likely due to their rapid corrosion and the associated drastic pH changes in their environment [177]. However, Mg–Li–(Al)–(REE) series alloys that undergo specific treatments often display low hemolysis rates and good cell viability. Furthermore, surface modifications such as Mg–OH, Mg–HF, Mg–P, and Mg–PA treatments can significantly reduce the hemolysis rates of magnesium alloys [162,171]. These results indicate that the hemocompatibility of magnesium alloys can be significantly improved through appropriate surface treatments. This underscores the critical role of material selection and surface modification in enhancing the biocompatibility of vascular stents during their design phase [166].

### 4.2. Cytocompatibility

Cytotoxicity testing is a critical step in assessing whether a material is toxic to cells, particularly in the development of cardiac stents. These materials must not exhibit toxicity toward endothelial cells, which form the blood vessel walls, or blood cells. Such tests are typically conducted in accordance with the ISO 10993-5 [161] standard, which includes direct and indirect contact testing methods. However, these standards were originally designed for non-degradable materials and may not be suitable for degradable materials, from which byproducts will be induced [178]. For instance, when assessing the cytotoxicity of magnesium-based alloys, it is recommended to use ten times the amount of extraction medium specified by the ISO standard to obtain reliable results [179]. Biocompatibility at the cellular level requires that biomaterials are non-toxic to endothelial cells, blood cells, and other relevant cell types, as any cytotoxicity could impair the function of the vessel wall and disrupt normal blood flow [180]. Therefore, cytotoxicity evaluations for stents often involve testing on endothelial cells, blood cells, or smooth muscle cells (SMCs), which provide a comprehensive understanding of the material’s effects on critical vascular functions. After determining the biocompatibility and safety of the candidate materials, decisions can be made regarding stent design and material selection [181].

To comprehensively evaluate the cytotoxicity of materials, researchers have employed a variety of cell lines to conduct in vitro experiments on zinc, magnesium, iron, and their biodegradable alloys. The majority of the cell lines used are derived from rodents, such as BALB/3T3 fibroblasts and CHO-K1 epithelial cells. Although these cell lines do not fully replicate the tissue environment surrounding stents, they are widely recommended for toxicological evaluations due to their ability to provide preliminary insights into the material’s potential toxicity [33]. However, since cardiac stents directly interact with the human vascular system, it is better to utilize human cell models that can represent the clinical environment of vascular stents. For instance, human endothelial cells (HUV-EC-C, CRL-1730), primary smooth muscle cells (SMCs), and adipose-derived stem cells (ADSCs) can accurately simulate the physiological environment, enabling a better evaluation of the materials’ performance and compatibility with the human body. Consequently, selecting appropriate cell types for cytotoxicity testing is not only vital for accurately assessing the biocompatibility of materials but also forms the foundation for ensuring the safety and efficacy of implantable devices in clinical applications.

**Figure 9:**
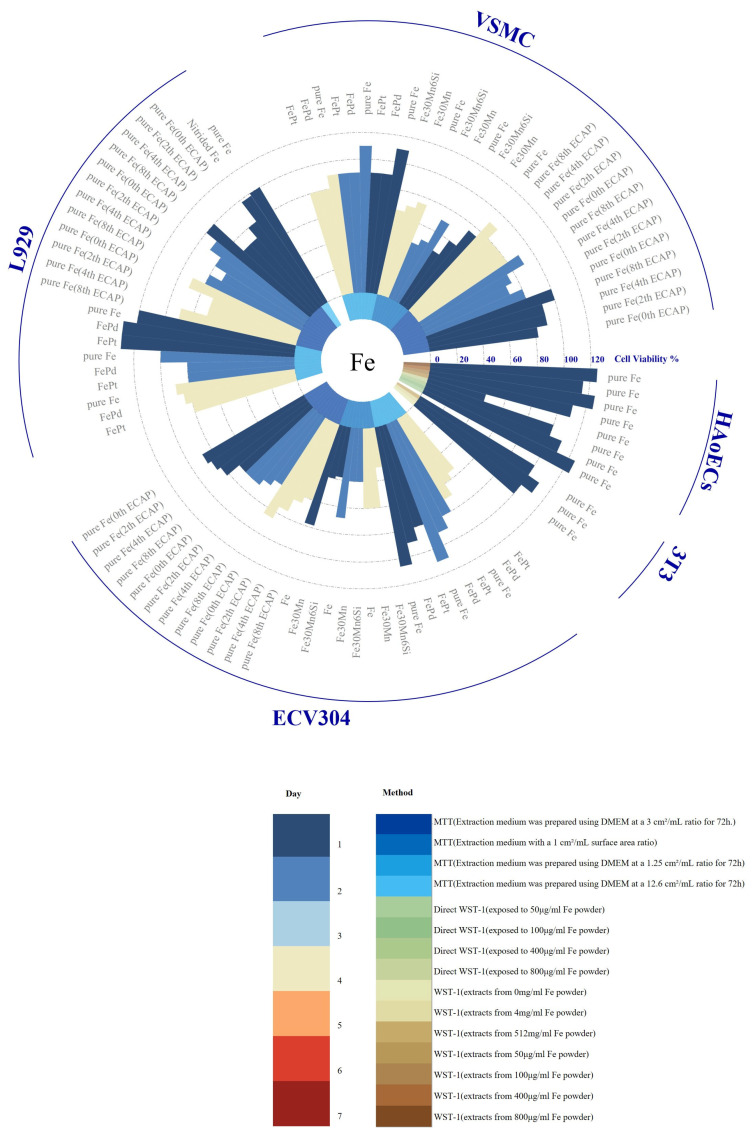
Cytotoxicity of Fe-based stents and their biodegradable alloys [110,115,167,182,183,184].

**Figure 10:**
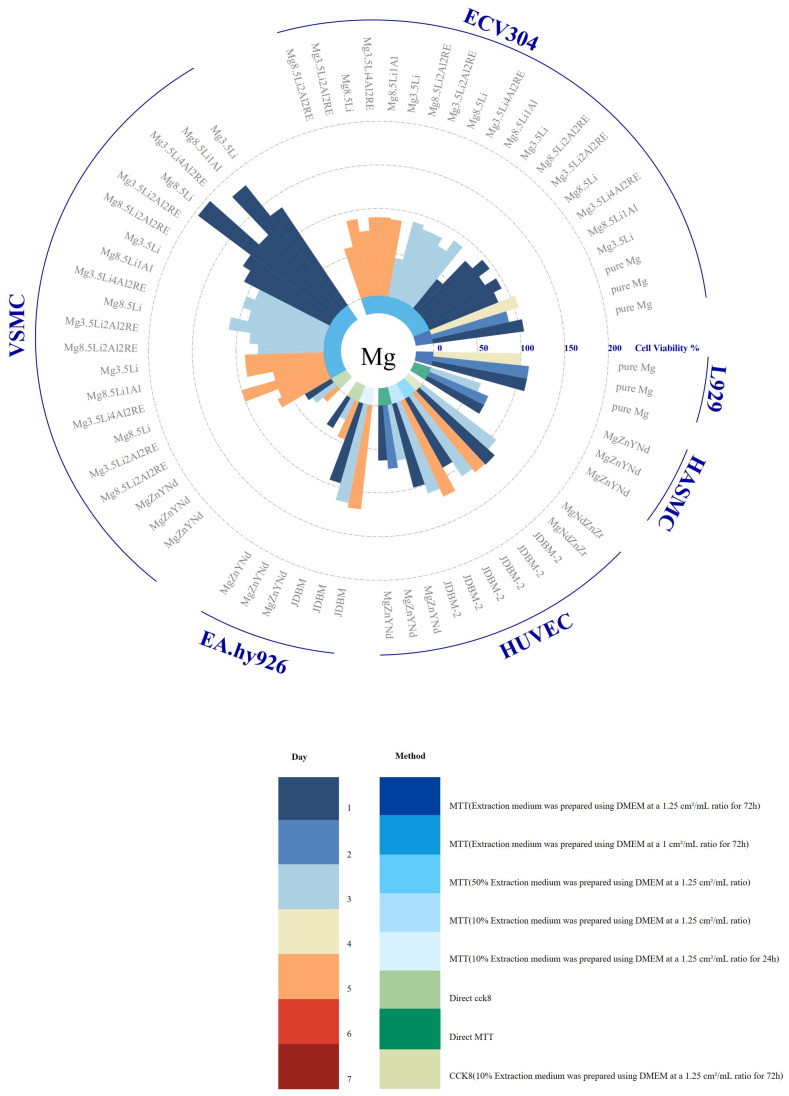
Cytotoxicity of Mg-based stents and their biodegradable alloys [79,85,174,185,186,187,188].

**Figure 11:**
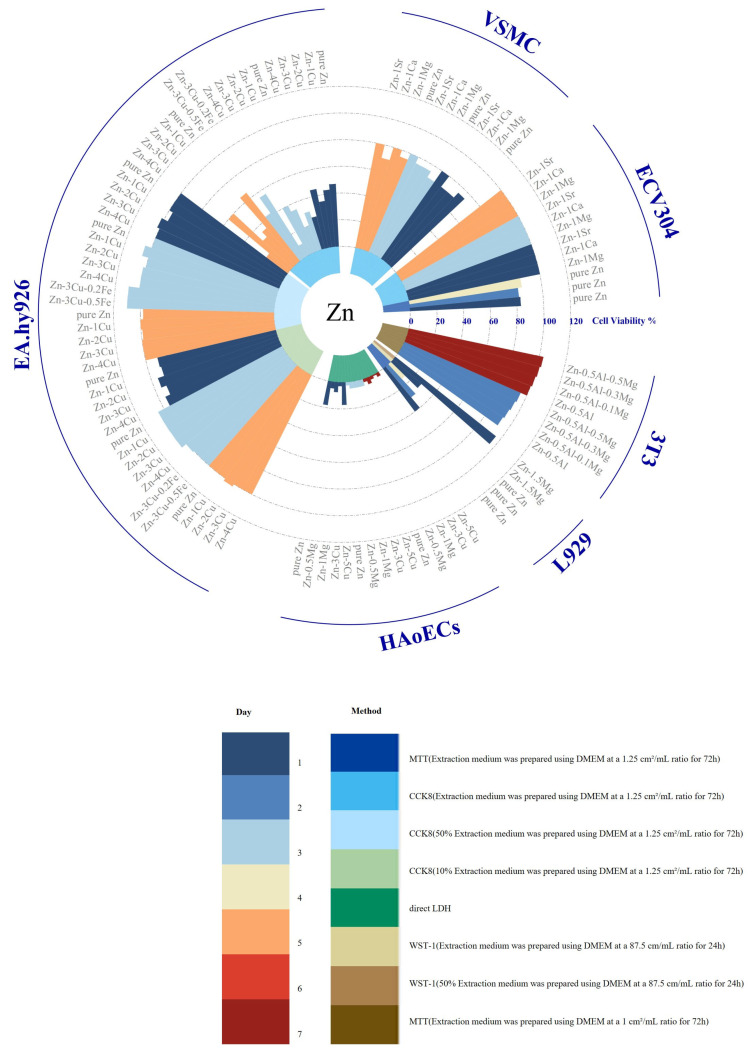
Cytotoxicity of Zn-based stents and their biodegradable alloys [87,132,168,176,185,189,190,191].

The three primary types of biodegradable metals—magnesium, iron, and zinc—have distinct advantages and limitations in terms of biocompatibility. A variety of cell lines have been used to evaluate the cytotoxicity of these biodegradable alloys via in vitro experiments, as shown in Figure 9, Figure 10 and Figure 11.

Magnesium-based materials are considered promising candidates for bioresorbable stents due to their excellent biocompatibility. However, exceptions exist; for instance, MgZnYNd has demonstrated significant cytotoxicity toward HASMC, HUVEC, EA.hy926, and VSMC in direct contact experiments. This toxicity may negatively impact endothelial cell proliferation and contribute to the development of intimal hyperplasia. Moreover, the rapid degradation of magnesium can result in high local concentrations of magnesium ions, potentially inducing coagulation or inflammatory reactions and delaying tissue remodeling. Additionally, magnesium degradation releases hydrogen; however, there is no direct evidence currently indicating any adverse effects [192]. Pure iron and manganese-free ferroalloys have shown good biocompatibility in indirect contact tests with various cell types, exhibiting no significant effects over co-culture durations [110,115,167,182,183,184]. However, direct contact with ferrous materials can induce intracellular oxidative stress and cytotoxicity [184]. Therefore, effective strategies for minimizing reactive oxygen species (ROS) generation are necessary. During degradation, the corrosion products of iron generate ROS, including highly toxic hydroxyl radicals (HO·), which can cause oxidative stress and cell death [33]. Zinc-based alloys exhibit a moderate degradation rate and good biocompatibility. The cellular response to zinc release is concentration-dependent: low zinc concentrations promote cell proliferation, adhesion, and migration, while higher concentrations inhibit these processes [193]. However, zinc materials often exhibit poor viability in indirect in vitro cell culture [190], primarily due to the absence of a cohesive protein layer on their surfaces [35]. Surface pretreatments, such as collagen modification or simulated body fluid (SBF) preincubation, have been shown to significantly enhance the cytocompatibility of zinc-based alloys, reducing the corrosion rate, improving cell adhesion, and enhancing metabolic activity [87].

Figure 9, Figure 10 and Figure 11 also show that the immersion test conditions of different studies are significantly different, which makes the research results lack comparability. Different experimental conditions, such as soaking time, extraction ratio, temperature, and solution pH, may lead to significant changes in degradation rates and products; therefore, it is critical to establish standardized in vitro assessment methods to ensure the reliability and reproducibility of research results. In addition, cytotoxicity testing is also affected by the interaction between a variety of factors, such as the type of alloy studied, the type of cells, the composition of the cell culture medium, the nature of the corrosion products, and dilution concentration. Changes in these factors may cause cells to react significantly, thereby affecting the results of cytotoxicity assessments. Additionally, the cytotoxicity studies in the figure mainly focus on exposure to the extract, assuming that the released soluble ions are the main source of cytotoxicity, while ignoring the possible cytotoxicity caused by insoluble degradation products. Studies have shown that the cytotoxicity of direct exposure to corrosion particles is significantly higher than that of exposure to extracts alone, suggesting the need to evaluate supernatants and degradation products separately to identify the specific components responsible for toxicity [182]. However, current cytotoxicity investigations predominantly focus on extract exposure, operating under the assumption that soluble ions constitute the primary cytotoxic source while neglecting potential toxicity from insoluble degradation products—a critical gap requiring methodological refinement. Additionally, a critical limitation of existing models lies in their exclusive use of healthy cell lines, whereas clinical applications target pathologically altered tissues. To enhance translational relevance, cytotoxicity assessments should prioritize disease-specific models, particularly those derived from patients with coronary artery disease or ApoE-deficient murine cells developing atherosclerosis. Such models would better replicate the pathophysiological microenvironment encountered during vascular stent implantation, thereby improving the clinical predictability of experimental outcomes [194].

### 4.3. In Vivo Studies

In vivo experiments on cardiac stents usually include animal experiments and clinical trials. The main purpose of animal studies is to preliminarily assess the feasibility and safety of bioresorbable stents. The scope of the study encompasses the delivery performance of the stent, systemic toxicity, local toxicity, the degradation characteristics of the stent in vivo, and the change in mechanical properties. The following factors must be considered when conducting animal studies on in vivo degradation: selection of test animal models, duration of animal testing, and the evaluation criteria. When selecting a test animal model, the metabolism, circulation, implantation site, body temperature, and other relevant factors should be considered based on the human physiological environment. The evaluation indexes of the target vessel site during the animal test generally include (1) morphological characteristics of the neointima and the degree of neointimal coverage of the stent struts; (2) the degree of endothelialization; (3) alterations in the middle and outer membranes; (4) structural integrity of the vessel wall; (5) inflammatory reactions and fibrosis in the neointima, middle, and outer membranes; and (6) stent degradation. Evaluation of degradation characteristics after stent implantation in animals mainly includes molecular weight reduction, changes in molecular weight distribution, mass loss, and mechanical changes (e.g., radial support).

The first human trials can be conducted only after the results of animal experiments have indicated sufficient safety and preliminary feasibility of the stent. The purpose of clinical trials is to evaluate whether the stent has the expected safety and efficacy. The safety indicators of stent clinical trials include death, myocardial infarction, and in-stent thrombosis. The postprocedural effectiveness index is the main observational measure of the implant and is used to evaluate the ability of coronary stents to maintain continuous vascular flow. The main factors influencing efficacy are stent restenosis, stent thrombogenicity, and stent structural failure. The indices for evaluating efficacy include Target Lesion Revascularization (TLR), Target Vessel Revascularization (TVR), and late lumen loss (Late Loss) within the stent or within the segment. The percentage of diameter stenosis can be used as an alternative index for evaluating efficacy. A composite index is a combination of indices that reflects the safety and effectiveness of a product, such as the Target Lesion Failure (TLF) index, which includes cardiac death, target vessel myocardial infarction, and target lesion revascularization. Composite indicators are usually chosen for evaluation in clinical trials.

#### 4.3.1. Progress in Clinical Trials of Mg-Based Metal Stents

A substantial number of clinical studies have been conducted on degradable magnesium alloy stents, including magnesium bare metal stents and drug-eluting magnesium alloy stents, as detailed in Table 12.

Erbel and Waksman et al. [195,201] reported the early and long-term results of the AMS-1 stent implanted in human coronary arteries. This clinical trial demonstrated that the pure magnesium stent exhibited good lumen gain during the early stages of implantation and completely degraded after 4 months [195]. There were no cases of myocardial infarction, subacute or late thrombosis, or death [201]. However, its negative remodeling and endothelial hyperplasia led to a high rate of in-stent lumen loss and target lesion hemodilution reconstruction 4 months after implantation.

To further address this problem, the drug-eluting magnesium alloy stent called DREAMS-1G was developed [196]. This stent improved the structural design and alloy composition and was coated with a polylactic acid–hydroxyglycolic acid copolymer (PLGA) drug-carrying coating and paclitaxel to inhibit endothelial hyperplasia based on AMS. Clinical studies using BIOSOLVE-1 showed that DREAMS-1G had lower late lumen loss than AMS (12 months (0.52 ± 0.39) mm vs. 4 months (1.08 ± 0.49)), and its clinically driven target lesion revascularization rate was also superior to that of AMS (4.7% vs. 26.7%) [196]. The 24-month and 36-month follow-up results of the BIOSOLVE-1 clinical trial confirmed the excellent safety and efficacy of DREAMS, with no deaths or stent thrombosis occurring over a 3-year period [197]. In addition, this magnesium alloy stent was completely degraded within 6 months [197].

The DREAMS 2G features a high-strength and flexible structural design, offering better flexural properties and higher radial support compared with the DREAMS 1G stent. It is coated with a polylactic acid–hydroxyacetic acid copolymer (PLLA) and carries the antiproliferative drug rapamycin. Clinical trials showed that DREAMS 2G decreased late lumen loss (in-segment late lumen loss: 0.83 mm (AMS-1) → 0.52 mm (DREAMS 1G) → 0.27 mm (DREAMS 2G); in-stent late lumen loss: 1.08 mm (AMS-1) → 0.65 mm (DREAMS 1G) → 0.44 mm DREAMS 2G) [64]. Corresponding to the reduction in late lumen loss, the clinically driven target lesion revascularization rate decreased to 1.7% at 4 months [64]. No definite or probable stent thrombosis occurred between 6 and 12 months after stent implantation [64]. The 3-year long-term follow-up of the DREAMS 2G Magmaris showed that angina resolved in 91.1% of patients and target lesion failure (TLF) occurred in only 8 (6.8%) patients, including 2 (1.7%) cardiac deaths, 1 (0.9%) target vessel myocardial infarction (TVMI), and 5 (4.3%) clinically driven target lesion revascularization (TLR) [198]. No stent thrombosis (ST) events occurred [98].

The subsequent BIOSOLVE-IV was an international study. In the first round, 1075 patients were involved, with a TLF of 4.3% at 12 months, including TLR (3.9%), cardiac deaths (0.2%), and TVMI (1.1%); the thrombus incidence was 0.5% [199]. In the second round, 2066 patients were included, with a TLF of 6.8% at 24 months, including TLR (6%), cardiac deaths (0.5%), and TVMI (1.6%); the thrombus incidence was 0.8%.

The biological safety of magnesium stents can be fully confirmed based on the results of the current clinical trials of magnesium stents; that is, the degradation products of magnesium stents have good biocompatibility, do not produce chronic inflammation in vivo, and have a low rate of endothelial hyperplasia. However, its degradation rate is too fast (the main stem of the stent loses its original stent shape 6 months after implantation, and 95% of the magnesium is converted 12 months after implantation), preventing it from satisfying the radial support force needed in vascular reconstruction. Stent collapse may also occur, triggering re-stenosis within the stent. In addition, the Magmaris stent is currently limited to the treatment of stable/unstable angina or non-ST-segment elevation myocardial infarction, and its safety has not been demonstrated in severely calcified, diffuse disease, hyperflexed, or angulated lesions [200].

#### 4.3.2. In Vivo Experimental Progress of Fe-Based Metal Stents

Some clinical studies on biodegradable iron alloy stents are included in Table 13, which also features stents made of pure iron and iron alloys.

Pure iron stents have demonstrated good biocompatibility in a variety of animal studies. In 2001, Peuster et al. evaluated the safety and performance of pure iron stents implanted in the right femoral artery of rabbits. No significant endothelial hyperplasia or inflammatory reaction was observed 18 months after implantation, and no systemic toxicity was induced [98]. In a 2006 porcine aortic implantation trial, it was shown that biodegradable iron stents had low thrombogenicity, and most of the stents remained intact after 1 year [99].

The difference in electrochemical properties of Fe and certain metals will result in the galvanic effect, increasing the corrosion rate. The Fe–Mn alloy is designed to take advantage of this. In a study of Fe–Mn, Fe–Mn–1Ag, and Fe–Mn–5Ag stent materials, the stent analogues were implanted in rats and removed 6 months later. They remained basically undegraded. Even though pure Fe, Fe–Mn, Fe–Mn1–Ag, and Fe–Mn–5Ag caused significant in vitro cytotoxicity against human fetal osteoblasts (hFOB) and mouse pre-osteoblasts (MC3T3-E1) cell lines, no adverse effects were observed in the 6-month in vivo experiments [202]. In another experiment in which Fe–Mn implants (0.5 wt%, 2.7 wt%, and 6.9 wt%) were evaluated in a mouse model, no obvious corrosion was detected in the implants after 9 months [116]. These results suggest that the current Fe–Mn alloy system requires improvement.

The nitrided iron-based stent was first developed by Lifetech Scientific (Shenzhen, China) Co., Ltd. and Biotyx Medical (Shenzhen, China) Co., Ltd. In 2017, Lin WJ et al. [108] compared the safety and performance of nitrided (0.074%wt) iron-based stents (70 μm thickness) with that of pure iron stents in the rabbit abdominal aorta. Endothelialization began on the seventh day of implantation, with mild inflammation. Both stents showed good long-term biocompatibility 36 months after implantation, with mass losses of 76.0 ± 8.5 wt% for nitrided iron-based stents and 44.2 ± 11.4 wt% for pure-iron stents. This suggests that the nitrided iron-based stents had a faster in vivo degradation rate. Nitrided iron-based stents with a thickness of 70 μm may take 4 to 5 years to completely corrode and 5 to 6 years to be completely bioabsorbed in a porcine coronary artery model [108].

By introducing a nanoscale Zn sacrificial layer between the nitrided iron-based and sirolimus drug-carrying PDLLA coatings, Danni Shen successfully designed Fe-based metal resorbable stents with a novel multilayer structure in 2021. This novel stent exhibited multistage biodegradation behavior in both rabbit abdominal aorta and human coronary artery. It maintained mechanical integrity in the initial stage and accelerated biodegradation in subsequent stages. Six months after implantation, the stent structures were embedded in the intima without significant recoil, and there was no intimal hyperplasia. Twenty-six months after implantation, the stents had degraded, but the degradation products were not completely absorbed. No significant lumen loss was observed at the 6-month and 26-month follow-ups [86]. In 2023, Gao RunL reported the results of the first 3-year clinical trial on this stent. Clinical data showed that the nitrided iron stent, with an ultra-thin thickness of 55–65 μm, can support vascular well, with a target lesion failure (TLF) rate of 2.2% 6 months after implantation, and a stabilized TLF rate of 6.7% 1–3 years after implantation. These results were comparable to the TLF rate of the Magmaris BIOSOLVE-IV (6.8%) at 24 months. No deaths, myocardial infarctions, or thrombotic events occurred throughout the follow-up period. There were no stenotic changes in the lumen during the 3-year follow-up period, and the stent did not show malapposition during degradation. It preliminarily demonstrated that this coronary stent has good medium-term safety and efficacy [66].

Both animal experiments and initial clinical studies have confirmed the in vivo safety of iron-based stents; however, the slow degradation rate of iron-based stents and the potential risk of cytotoxicity from the slow metabolic process of their degradation products limit their clinical application [203]. Therefore, increasing the degradation rate of Fe stents and promoting the metabolism of degradation products are critical for clinical application.

#### 4.3.3. In Vivo Experimental Progress on Zn-Based Metal Stents

A variety of zinc-based alloys, including pure Zn, Zn-Mg, and Zn-Sr, have been implanted into animals in studies examining biodegradable zinc-based alloys as vascular stent materials. Their biocompatibility and degradation behaviors are detailed in Table 14.

Since the standard electrode potential of Zn (−0.763 V) lies between that of Mg (−2.37 V) and Fe (−0.44 V), the degradation rate of pure Zn should be between that of Mg and Fe. The in vivo experiments confirmed this theory. It was found that pure Zn filaments uniformly degrade in rat aorta, with a degradation rate very close to the benchmark value (20 μm/a) for ideal bioresorbable stents. These pure Zn filaments, with diameters of 0.275 mm, largely retained their original shapes for 4 months, after which their degradation accelerated with time. Yang et al. also found that pure Zn stents retained their mechanical integrity 6 months after implantation, and the volume decreased by 41.75 ± 29.72% 12 months after implantation. This degradation behavior ensured that the stents were degraded and metabolized after vascular remodeling, and thus, conducive to the timely restoration of the diastolic function of the vessel wall. In addition, pure Zn material showed good biocompatibility in the abdominal aorta of both rats and rabbits, i.e., no significant inflammatory reaction, local necrosis, or endothelial hyperplasia occurred within 6 months of implantation, tissue regeneration occurred in the original location of the partially degraded stent, and the density of smooth muscle cells in the location neighboring the implanted stent was low, effectively avoiding restenosis after stent implantation. Neointimal coverage was observed 1 month after implantation of a pure Zn stent in the rabbit abdominal aorta, suggesting rapid endothelialization; no corrosive degradation products were detected 12 months after implantation, no thrombosis formed at the site of implantation, and there was no significant endothelial hyperplasia or loss of lumen [203,205].

The above studies have shown that pure Zn exhibits good biocompatibility and therapeutic properties, but with a greater corrosion rate; however, its mechanical properties are poor and do not meet the requirements for clinical application. Alloying and plastic deformation of Zn-based materials can lead to improved mechanical behavior; however, their in vivo degradation rates and biocompatibility might be affected. Li et al. investigated the in vivo degradation behaviors of the binary alloys Zn–Mg, Zn–1Ca, and Zn–1Sr in rats and found that the incorporation of these three alloying elements resulted in an acceleration of the degradation of the materials [176].

Current in vivo experiments have shown that Zn alloys, specifically Zn–0.1Li and Zn–0.8Cu, show better corrosion resistance compared to pure Zn. Zn–0.1Li alloy wire degraded at a rate of ~0.02 mm/year at 6.5 months and ~0.05 mm/year at 12 months in the abdominal aorta of rats. The corrosion volume of Zn–Li at 6 months was only slightly less than that of 4N (99.99%) Zn, but both retained ~70% of their original size after 12 months [206].

The stent volume retention of the Zn–0.8Cu stent was 28 ± 13% after 24 months of implantation in porcine coronary arteries [207].

Animal experiments have shown that pure Zn and Zn-based alloys have potential for clinical applications. Among them, pure zinc has a suitable degradation rate and good biocompatibility, but its mechanical properties are poor. Zn-based alloys have better mechanical properties, but their corrosion resistance and biocompatibility need further verification.

## 5. Advances in Stent Structure Research

**Figure 12:**
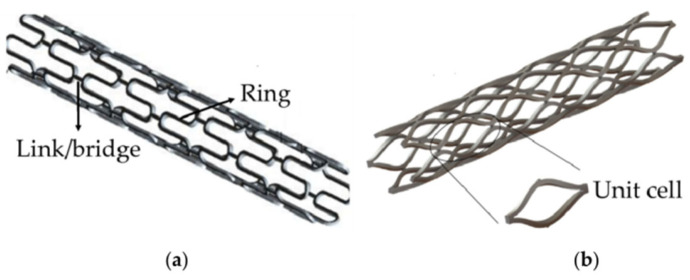
Two types of vascular stent designs [217]: (**a**) link stent [215]; (**b**) RVE/RUC stent [216]. Reprinted from Ref. [215], licensed under CC-BY. (**b**) Reprinted from Ref. [216], Copyright 2018 Elsevier.

The choice of materials for vascular stents is governed by various factors, including the need for sufficient radial strength to consistently support the vessel, non-toxicity, and an efficient manufacturing process. To address challenges associated with the mechanical properties and degradation rates of degradable heart stents, the design of the stent structure is currently considered pivotal [210,211]. Increased radial stiffness, improved axial flexibility, reduced foreshortening, and enhanced uniformity of tissue stress can prevent the occurrence of the “Dogbone” effect. Additionally, proper structural design can optimize the efficiency of drug release [212,213,214]. Two main types of support structures are currently available on the market. The first type consists of interconnected rings and links, as depicted in Figure 12a [215]. The second type is formed via the direct connection and arrangement of Representative Volume Elements (RVEs)/Representative Unit Cells (RUCs), illustrated in Figure 12b [216].

For the first type of stent structure, the ring mainly facilitates radial expansion and provides vascular support, while the links connect the rings axially to ensure the overall axial flexibility of the stent [218]. Studies focusing on link vascular stents have found that the geometric characteristics of the connecting parts are crucial in determining their mechanical properties [219,220,221,222,223,224]. Optimizing the shapes of these connections and their specific geometric parameters will enhance the mechanical performance and structural properties of stents. Among the various types of links in existing vascular stents—such as types I, C, L, N, V, S, U, and W—optimized links include JS, OCS, CCS, and asymmetric versions of traditional links. Table 15 compares the mechanical properties of different link types as determined in various experiments.

For the second type of stent structure, RVE/RUC, optimization mainly involves designing the cell and analyzing the performance of the resultant stent [229,230]. Current RVE vascular stent structures include Diamond, Re-entrant Auxetic, Hybrid A, Hybrid C, and Chevron B. Structures composed of materials with negative Poisson ratios (which expand or contract during stretching or compression), such as Re-entrant, Chiral, and Antichiral Re-entrant, are noted. Innovative types are Arrowed brackets and those with non-uniform cell sizes. Table 16 summarizes the mechanical properties of stents with different RVE structures reported by various research teams. It is worth noting that achieving both high strength and excellent flexibility in a single structure remains a challenge. Notably, in the field of innovative stent synthesis, the Ameer team [231,232,233] successfully synthesized a new Arrowed biodegradable polymer stent using a novel micro-continuous liquid interface fabrication technique, achieving unsupported printing. This study significantly simplified the post-processing steps of vascular stents, optimized the efficiency of the production process, and proposed a new approach for processing and synthesizing metal stents.

**Figure 13:**
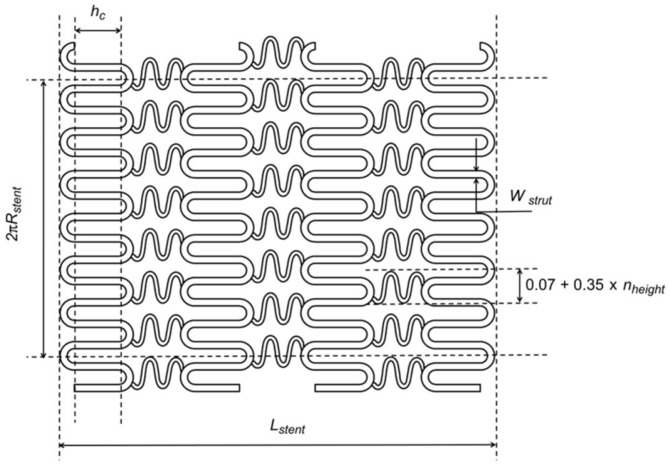
CYPHER stent parameterization. Reprinted with permission from Ref. [239]. Copyright 2011 Elsevier.

**Figure 14:**
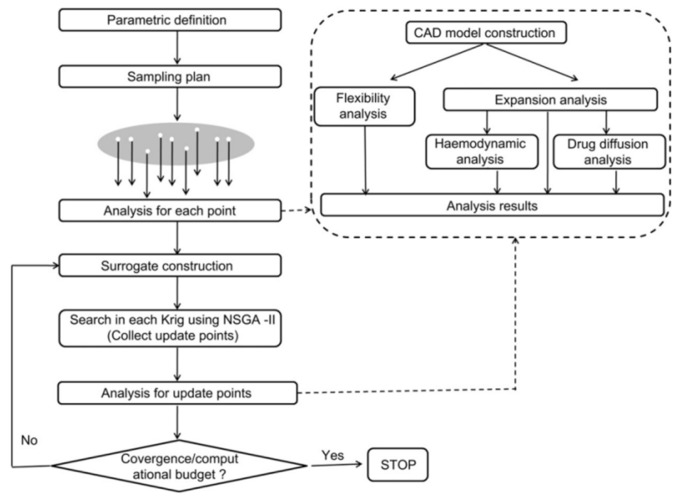
Flow chart detailing the optimization methodology. Reprinted with permission from Ref. [239]. Copyright 2011 Elsevier.

Studies on stent structural designs often focus on a single parameter, overlooking the interplay and balance among multiple variables. Further optimization should consider the influence of multiple geometric parameters on the performance of known stent structures. For instance, enhancing one performance metric might compromise another—increasing flexibility could elevate volumetric mean stress, while reducing hemodynamic interference might disrupt the uniform distribution of drugs. Sanjay Pant et al. [239] adopted a multi-objective and multidisciplinary approach to optimize the design of coronary artery stents, specifically the widely known CYPHER (Cordis Corporation, Johnson & Johnson co.) type stent. Through parametric design, they explored the effects of three geometric parameters of the stent (longitudinal length of the circumferential rings: $h_{c}$, circumferential strut width: $W_{Strut}$, and height of the links: $n_{hight}$, illustrated in Figure 13) on its performance. Performance metrics were measured through computational simulations, addressing stent structural deformation, hemodynamics, drug distribution, and response to external forces. Using the non-dominated sorting genetic algorithm (NSGA-II) and multi-objective agent modeling, the study revealed trade-offs between different design objectives. The study highlighted the essential concept of stent structure optimization as shown in Figure 14, providing valuable insights for achieving a more efficient and safer stent design. This method primarily uses finite element analysis (FEA) to evaluate the performance of the stent structure. First, a CAD model of the stent is constructed, and key geometric parameters for adjustment are identified. Material properties are then defined. The model is subsequently discretized into a number of finite elements, which can include triangles, quadrilaterals, tetrahedra, or hexahedra. Realistic boundary conditions and loads are applied to simulate various test scenarios, such as the Flexibility Test and Expansion Test. By solving the corresponding mathematical model—typically a system of matrix equations—the structural responses at each node, such as displacement and stress, can be obtained. Finally, post-processing is performed to visualize the results, generating displacement maps, stress distribution plots, and other outputs to assist in evaluating the structural performance. This methodology is also applicable to stents with non-uniform cell sizes. Torki et al. [240] optimized a stent model through parameter optimization and simulated its performance in an artery with 49% plaque heterogeneity. The results of finite element analysis revealed a 74% increase in the internal area of the artery cross-section, indicating an equivalent increase in blood flow.

**Figure 15:**
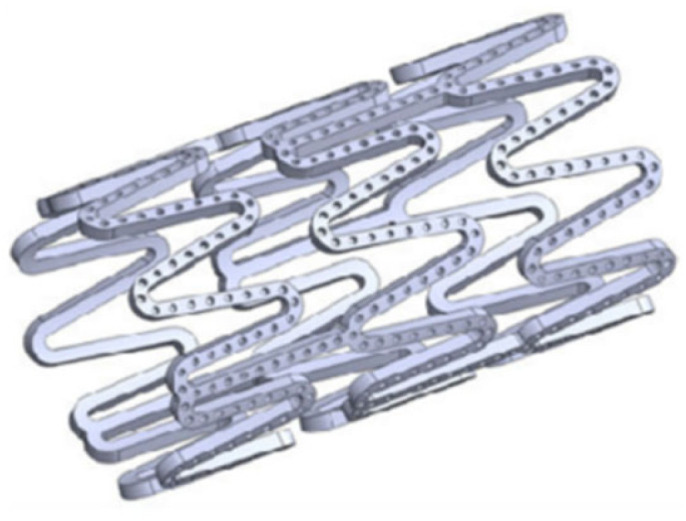
Structure of depot stent [241]. Reprinted with permission from Ref. [241]. Copyright 2012 Taylor & Francis Ltd.

Stents are generally coated with anti-proliferative drugs to effectively inhibit intimal hyperplasia, prevent early inflammatory responses after implantation, and enhance the rate of vascular restenosis, TLR, and TVR. However, in the current application of vascular stents, the drug release rate is often too slow, and carrier detachment can impact the long-term therapeutic effect and patient safety. Thus, when designing the stent structure, it is crucial to devise a configuration that can fully release the drug and stabilize the carrier. Hsiao et al. proposed an innovative solution by developing a stent with a surface micro-tank structure, depicted in Figure 15 [241]. By adjusting the size and distribution of the microtanks on the stent, the amount and rate of drug release can be precisely controlled. It can be tailored to fit various clinical needs and ensure maximum drug capacity. This configuration can carry more drugs than existing drug-eluting stents, albeit with trade-offs in key clinical attributes such as radial strength and fatigue durability.

**Figure 16:**
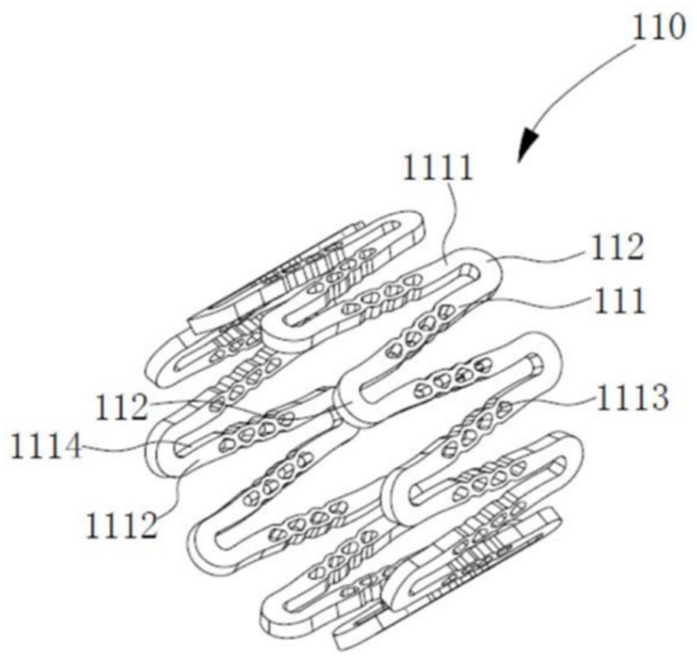
A ring structure with multiple through-holes designed by Lifetech Scientific [247]. Reprinted from Ref. [247].

Moreover, structural design may modify degradation rates. As discussed earlier (Section 3.1.1 and Section 3.2.1), the issues associated with the slow degradation rate of ferroalloys and the fast degradation rate of magnesium alloys have been identified. Several studies have attempted to address these challenges through structural optimization. According to Grogan et al. [242], the problem of excessive degradation rate for magnesium alloys is linked to pitting caused by local stress concentration, which significantly impairs the mechanical properties and the effective lifespan of Mg-based stents. Current research focuses on reducing stress concentration in stents during expansion and crimping to prevent local stress corrosion. Wu et al. [243,244] proposed a method for mitigating the impact of stress corrosion degradation by minimizing the maximum principal stress of the stent during expansion and deformation. Finite element method simulations were conducted to model the degradation of the magnesium alloy stent (based on uniform degradation and stress corrosion degradation mechanisms), leading to an optimized structure. During the optimization of the JDBM sine wave stent structure, Chen et al. [245] introduced a convex platform structure to facilitate the parallel compact arrangement of supports in the curly state of the stent, thus fully aligning the mechanical properties of the magnesium alloy stent. This innovative shape optimization strategy effectively dispersed the residual stress distribution during the deformation of the support and controlled the deformation behavior of the support. Compared to the traditional sine-wave stent design, the shape-optimized stent not only significantly reduced the “dog bone” effect (22.1% vs. 28.3%) and axial shortening (0.6% vs. 2.7%) but also enhanced the radial support strength (96.7 kPa vs. 88.8 kPa). At the same time, the high residual stress area was also significantly reduced (0.68% vs. 4.12%). For iron alloys, the low degradation rate is a big challenge. Lifetech Scientific (Shenzhen, China) Co., Ltd. has undertaken extensive optimization of the structural design of the IBS stent, which is made from an iron alloy. First, the absorbable stent has a lower volume ratio of the matrix per unit vascular area (two adjacent ring structures form a closed collateral unit with the link unit) under the condition that the radial support strength satisfies the clinical application requirement [246]. Thus, shorter corrosion and absorption periods can be obtained using the same material. Additionally, a corrosion inhibition layer and a corrosion promotion layer are coated on the support matrix, and several through holes are present on the ring structure, as depicted in Figure 16 [247]. The thickness of the corrosion-inhibiting layer at the through hole is smaller than that of other parts, while the thickness of the corrosion-promoting layer at the through hole is larger than that of other parts. The structural design is conducive to maintaining sufficient mechanical support properties during the repair of the lesion, and it degrades quickly after the repair is completed, thereby releasing the bondage on the vascular wall. Moreover, the pro-corrosion coating is also distributed in the axial link [246,248] and contains pro-corrosion substances such as degradable polymers and degradable polymer antioxidants. The coating promotes the corrosion of the axial link earlier than the ring structure, allowing the support to be orderly deconstructed axially to form multiple independent waveform ring structures, thereby improving the bending performance and reducing the narrowing rate of the stent.

Regardless of the sophistication of their structural designs, stents struggle to perfectly fit all patients with varying vascular geometries. Stent malapposition and poor mismatch can lead to endothelial injury and alterations in hemodynamics, potentially causing adverse effects such as thrombosis and stent restenosis, thus diminishing their therapeutic efficacy and increasing discomfort and cost for patients [249,250]. To address this issue, an innovative solution—patient-specific vascular stents—is proposed. These stents are customized based on the unique vascular morphology of each patient, using high-precision medical imaging techniques such as Computed Tomography (CT), X-ray Angiograms, and Magnetic Resonance Imaging (MRI) to capture detailed three-dimensional structures of the patient’s arteries [251]. Combining the captured images and advanced Computer-Aided Design (CAD) technologies, a personalized stent that conforms precisely to the patient’s vascular anatomy can be produced. In this way, the unnecessary contact and friction between stent and vessel wall are reduced, lowering the risk of vascular damage and thrombus while also allowing the drug coating to be tailored to the patient’s specific condition, thereby enhancing the prevention of restenosis. Here, 3D printing technology plays an important role, enabling accurate and swift fabrication of complex stent structures. Although 3D printing is currently primarily utilized for creating polymer stents [252], its application to metal-based stents is under exploration. As demonstrated by Han and Lu [253], by designing vascular stents with a non-uniform Poisson ratio, these customized stents can better accommodate the patient’s vascular curvature and effectively minimize the stress concentration between the stent and the vascular wall, significantly reducing the risk of vascular intimal thickening and in-stent restenosis (ISR).

## 6. Summary and Outlook

In general, Mg-based, Fe-based, and Zn-based alloys are the most widely used metals in biodegradable stents [254]. Table 17 shows the most significant advantages and concerns associated with the use of these alloys in stents.

On this basis, research focuses on material properties and selection, stent degradation, biosafety, and stent structures. Based on the findings of this study, we provide a summary of the key points and related outlook.

### 6.1. Experimental Conditions for Dynamic Corrosion In Vitro

In vitro experiments cannot fully replicate the complex physiological conditions in the human body, resulting in discrepancies in corrosion behavior between in vitro and in vivo environments. Over time, the simulated environment may further deviate from in vivo conditions. For example, after the endothelialization process is complete, the contact area between the stent material and flowing blood decreases, reducing the impact of blood shear stress on the corrosion process and altering the hemodynamic environment.

To enhance the accuracy of simulation experiments, it is essential to account for a broader range of experimental conditions influencing metal corrosion behavior. When designing in vitro corrosion experiments, it is advisable to progressively adjust the experimental setup to better align with the temporal changes in the internal physiological environment. Hemodynamic parameters corresponding to the implantation site can be utilized to simulate body fluids prior to the completion of endothelialization, during which fluid flushing predominantly governs the interaction between blood and the stent. After endothelialization, the influence of fluid shear stress diminishes, and the parameters of the in vitro corrosion experiment can be adjusted to reflect those of the extracellular fluid environment. This approach may provide more reliable insights into the degradation behavior of biodegradable metal materials and their potential biological effects on the human body.

### 6.2. Optimization of Biodegradable Alloy Materials

- Magnesium alloy system: Relative to ideal stents, magnesium alloy stents exhibit weaker mechanical properties and a rapid degradation rate. Therefore, the development of biodegradable magnesium alloy stents should consider optimizing the stent size and controlling the degradation rate while maintaining their radial support. Currently, surface modification represents the primary strategy for enhancing the corrosion resistance of magnesium alloy stents, with coating application being the most prevalent method. However, given that the stent expands during implantation, the coating may fracture or detach due to disparities in mechanical properties between the coating and the base material, substantially increasing the risk during and post-surgery. To overcome this limitation, it is crucial to explore novel material systems, composite structures, and coating methods that can prevent the coated layer from separating from the metal base during stent implantation.
- Ferroalloy system: Iron-based stents primarily contend with a slow degradation rate, which can lead to late thrombosis and chronic inflammation, and may also interfere with MRI processes.
- Zinc alloy system: Zinc alloys degrade at a moderate rate, yet their overall strength and ductility remain inferior to the aforementioned materials. The research and development of biodegradable zinc alloy stents has received attention in recent years.

Alloying can enhance the mechanical properties and corrosion resistance of metal-based stents to some extent. However, adding new elements can lead to ambiguous biocompatibility due to the formation of new phases and corrosion products, necessitating careful consideration of the changes in properties. During the exploration of film modification methods, it is necessary to analyze the adhesion between the coating and the substrate when the stent is expanded and compressed.

### 6.3. Biocompatibility of Degradable Alloy Materials

Magnesium-based, iron-based, and zinc-based materials exhibit good biocompatibility; however, some aspects require improvement. The rapid and local degradation of magnesium restricts the application of magnesium-based materials; additionally, the hydrogen gas and corrosion products released during the degradation process may affect the surrounding tissues. Similarly, direct contact with iron-based materials can lead to the release of reactive oxygen species (ROS), which may cause cell damage and inflammatory responses. Moreover, iron-based stents degrade slowly. The main corrosion products of iron-based stents (Fe_3_O_4_, Fe_2_O_3_, FeOOH) dissolve slowly, which significantly reduces systemic toxicity; nevertheless, the 3–4-year degradation cycle of iron-based stents still poses a potential risk of cytotoxicity. Zinc-based materials have a moderate rate of degradation. However, cells generally lack viability in vitro cell cultures. Experiments have shown that pure Zn can be safely degraded in the body over a prolonged period. However, there are few in vivo experiments on Zn and zinc-based stents. More animal and clinical experiments are required to further investigate their impacts on the human body.

Overall, the test conditions for the biocompatibility studies of existing degradable metals vary significantly (as shown in Figure 9, Figure 10 and Figure 11). To enhance comparability across different studies, it is recommended that consistent test conditions and evaluation criteria be adopted when conducting cytotoxicity tests. Secondly, the current cytotoxicity studies mainly focus on exposure to extracts. Soluble ions are usually considered the main source of cytotoxicity, whereas cytotoxicity caused by insoluble degradation products is often overlooked. Therefore, when evaluating cytotoxicity, a comprehensive assessment of both soluble and insoluble degradation products should be carried out. In addition to detecting soluble ions in the extracts, direct contact tests can also be conducted. By directly culturing cells on the material surface or degradation products, the specific components that cause toxicity can be identified. Thirdly, the surrounding local environment undergoes constant change following the implantation of degradable metal stents. To evaluate the biocompatibility of materials under conditions closer to the actual in vivo situation, a series of experiments simulating the dynamic in vivo environment may be used in the early and middle stages. For example, co-culturing endothelial cells and smooth muscle cells of patients with coronary artery disease with non-corroded materials to simulate the initial wound healing stage, followed by co-culturing soluble and insoluble degradation products with endothelial cells, macrophages, and fibroblasts to simulate the interactions occurring during the neointimal hyperplasia process in order to determine the impact of materials on the repair and restenosis processes after vascular injury.

### 6.4. Stent Structure

Stent architecture optimization improves the mechanical properties and degradation rates of biodegradable stents, enhancing radial stiffness, axial flexibility, and drug release efficiency. However, designing stents that perfectly fit the diverse vascular morphologies of patients remains a challenge. Customized stents tailored to individual patients can improve adherence to blood vessel walls, offering better treatment for conditions such as atherosclerosis. Advanced medical imaging techniques such as Optical Coherence Tomography (OCT), Computed Tomography Angiography (CTA), Conventional Coronary Angiography (CCA), X-ray angiography, and Magnetic Resonance Imaging (MRI) enable the precise capture of vascular structures, guiding the design of personalized stents. For example, Morlacchi et al. reconstructed two patient-specific coronary bifurcations using CCA and CTA and simulated stent deployment through finite element analysis with high accuracy, offering a foundation for patient-specific design and subsequent manufacturing [255]. Three-dimensional printing technology is then used for accurate and rapid fabrication of stents that match a patient’s vascular anatomy.

Additionally, the aggregation and sharing of extensive individual medical records, combined with the use of AI algorithms, can significantly contribute to the future development of personalized stent designs. By leveraging large datasets from diverse patient populations, AI can identify patterns and predict optimal stent configurations based on specific anatomical and pathological characteristics. This approach not only accelerates the design process but also ensures a higher degree of precision in stent customization.

## Figures and Tables

**Table 1:** Summary of material criteria and constraints for a biodegradable stent [15].

Criterion	Constraint
Biodegradation	Mechanical integrity for 3~6 months; Full absorption in 12~24 months
Biocompatibility	Non-toxic and non-inflammatory; No allergenic potential; No harmful release or retention of particles
Mechanical properties	Yield strength > 200 MPa, Ultimate tensile strength > 300 MPa; Yield strength/elastic modulus ratio > 0.16; Elongation to failure > 15~18%; Elastic recoil on expansion < 4%
Microstructure	Homogeneous and approximately isotropic
Small grain size	<30 um
Corrosion rate	Penetration rate < 0.02 mm∙a^−1^

Adapted with permission from Ref. [15]. Copyright 2016 Wiley.

**Table 2:** Important constituents and physical characteristics of the extracellular fluid [26].

	Normal Value	Normal Range	Approximate Short-Term Nonlethal Limit	Unit
Sodium ion	142	138–146	115–175	mmol/L
Potassium ion	4.2	3.8–5.0	1.5–9.0	mmol/L
Calcium ion	1.2	1.0–1.4	0.5–2.0	mmol/L
Chloride ion	108	103–112	70–130	mmol/L
Bicarbonate ion	28	24–32	8–45	mmol/L

Reprinted with permission from Ref. [26]. Copyright 2011 Elsevier.

**Table 3:** Summary of the composition of diverse simulated body fluids.

Solution	Test	Composition	Ref.
Hank’s	Immersion Electrochemical	8.0 g/L NaCl,	[27]
		0.4 g/L KCl,	
		0.14 g/L CaCl_2_,	
		0.35 g/L NaHCO_3_,	
		1.0 g/L C_6_H_6_O_6_ (glucose),	
		0.2 g/L MgSO_4_·7H_2_O,	
		0.1 g/L KH_2_PO_4_·H_2_O,	
		0.06 g/L Na_2_HPO_4_·7H_2_O	
Simulated Body Fluid (SBF)	Immersion Electrochemical	142.0 (mmol/L) Na^+^	[28]
		5.0 (mmol/L) K^+^	
		2.5 (mmol/L) Ca^2+^	
		1.5 (mmol/L) Mg^2+^	
		147.8 (mmol/L) Cl^−^	
		4.2 (mmol/L) HCO_3_^−^	
		0.5 (mmol/L) SO_4_^2−^	
		1.0 (mmol/L) HPO_4_^2−^	
Modified Simulated Body Fluid (m-SBF)	Immersion Electrochemical	5.403 g/L NaCl,	[29]
		0.504 g/L NaHCO_3_,	
		0.426 g/L Na_2_CO_3_,	
		0.225 g/L KCl,	
		0.230 g/L K_2_HPO_4_·3H_2_O,	
		0.311 g/L MgCl·6H_2_O,	
		100 mL 0.2 mol/L NaOH,	
		17.892 g/L HEPES,	
		0.293 g/L CaCl_2_,	
		0.072 g/L Na_2_SO_4_	
PBS	Immersion Electrochemical	0.20 g/L KCl,	[30]
		0.20 g/L KH_2_PO_4_,	
		8.00 g/L NaCl,	
		1.15 g/L Na_2_HPO_4_	
Modified HBSS	Immersion Electrochemical	8.0 g/L NaCl,	[31]
		0.4 g/L KCl,	
		0.14 g/L CaCl_2_,	
		0.35 g/L NaHCO_3_,	
		1.0 g/L C_6_H_6_O_6_ (glucose),	
		0.2 g/L MgSO_4_·7H _2_O,	
		0.1 g/L KH_2_PO_4_·H_2_O,	
		0.06 g/L Na_2_HPO_4_·7H_2_O	
		4-(2-hydroxyethyl)-1-piperazineethanesulfonic acid	
DMEM	Immersion	Dulbecco’s modified Eagle’s medium	[32]
Culture medium	Immersion	Contains inorganic ions, organic compounds, and proteins	[33]
Human plasma	Electrochemical	Contains inorganic ions, organic compounds, and proteins	[34]
Whole blood	Electrochemical	Contains inorganic ions, organic compounds, and proteins	[35]

**Table 4:** Coronary hemodynamic parameters.

Parameter	Value	Unit	Ref.
Blood flow range	200–250 (About 4 to 5 percent of the total cardiac output.)	mL/min	[38]
Pressure	100–180	mmHg	[38]
Viscosity	3.5	N·s/m^2^	[39]
Diameter (left main artery)	4.5 ± 0.5	mm	[39]
Diameter (right coronary artery)	3.9 ± 0.6 and 2.8 ± 0.5	mm	[38]

**Table 6:** Differences in the mechanical properties of different Mg alloys.

Material	YS (Mpa)	UTS (Mpa)	EL (%)	HV
Mg-3Al-1Zn (AZ31) [57,58]	172	-	16	-
Mg-2-1Zn-0.2Zr (JDBM) [59,60]	123–220	267	26–48.8	-
Mg-4Y-3RE (WE43) [61,62,63]	113	340–440	10–21	102–114
Mg-2Zn-1RE-B (ZE21B) [64]	196	298	20	-
Mg-2Zn-1Mn (ZM21) [65,66,67]	340	353	11.5	-
Mg-4Zn-1Y [51]	240	330	20.4	-

**Table 7:** Effect of nitriding on the mechanical properties of iron stents (tensile strength, radial strength, stiffness, and microhardness) [102].

	σ_0.2_ (MPa)	Tensile Strength (MPa)	Radial Strength (kPa)	Stent Stiffness (kN/m)	Microhardness (HV)
Pure iron stents	236.27 ± 22.40	342.90 ± 44.18	59.07 ± 3.69	37.87 ± 2.93	154.92 ± 5.24
Nitrided iron stents	561.41 ± 30.99	614.35 ± 29.03	92.61 ± 5.26	52.95 ± 3.96	287.06 ± 8.67
*P*	<0.01	<0.01	<0.01	<0.01	<0.01

HV, Vickers hardness.

**Table 8:** Effect of nitriding on in vitro corrosion rate of iron stents [102].

	$V_{corr}^{a}(V)$	$I_{corr}^{b}$ (µA cm^−2^)	$v_{corr}^{c}$ (mm year^−1^)
Pure iron stents	−439 ± 10	10.887 ± 0.715	0.127 ± 0.008
Nitrided iron stents	−405 ± 39	19.365 ± 1.731	0.225 ± 0.020
*P*	>0.05	<0.01	<0.01

^a^ Corrosion potential; ^b^ Corrosion current density; ^c^ Corrosion rate; Table 7, Figure 7, and Table 8 are reprinted with permission from Ref. [102]. Copyright 2012 Springer Nature.

**Table 9:** Mechanical properties of ferroalloys after introducing intermetallic phases [113].

Designation	YS(MPa)	UTS (MPa)	*ε_u_* (%)	*ε_f_* (%)	H (HV10)
Fe (Armco)	250	300	19.5	37.5	85 ± 1
Fe (sht)	700	900	3.5	9.5	403 ± 2
Fe-10Mn (sht)	NA *	NA *	NA *	NA *	428 ± 6
Fe-10Mn (ht 1)	800	1400	9.5	14.0	384 ± 5
Fe-10Mn (ht 2)	650	1300	9.0	14.0	374 ± 7
Fe-10Mn-1Pd (sht)	950	1500	2.0	2.0	432 ± 8
Fe-10Mn-1Pd (ht 1)	900	1550	6.5	7.0	437 ± 3
Fe-10Mn-1Pd (ht 2)	850	1450	8.0	11.0	376 ± 6

* Data not available. Reprinted with permission from Ref. [113]. Copyright 2010 Elsevier.

**Table 10:** Differences in the mechanical properties of different Zn alloys.

Alloys	YS (Mpa)	UTS (Mpa)	EL (%)	Hardness
Zn-0.08Mg [129]	221	339	40	103
Zn-0.8Mg [130]	203	301	15	80–90
Zn-1Mg [131,132]	316–383	435–482	23–35	-
Zn-1.2Mg [133]	219.61	362.64	21.31	96.01
Zn-1Mg-0.1Ca [134]	209.04	331.51	35.43	110–125
Zn-2Al-0.4Li [135]	352.2	405.8	31.6	-
Zn-4Al-0.2Li [135]	362.7	382.2	33.3	-
Zn-4Al-0.4Li [135]	399.3	414.6	25.6	-
Zn-4Al-0.6Li [135]	429.6	451.4	46.3	-
Zn-6Al-0.4Li [135]	335.6	432.6	42.7	-
Zn-2Al-2Cu-0.6Li [136]	260	402	44	-
Zn-2Al-2Cu-0.8Li [136]	371	445	47	-
Zn-2Al-4Cu-0.6Li [136]	404	535	32	-
Zn-2Al-4Cu-0.8Li [136]	407	536	17	-
Zn-2Cu-0.3Li [137]	226.55	333.49	26	124.59
Zn-3Cu-0.5Fe [138]	269	302	24.7	-
Zn-3.5Cu-0.3Li [137]	231.63	382.8	18.33	128.79
Zn-4Cu-0.02Li [139]	256	342	39.8	-
Zn-5Cu-0.3Li [137]	272.87	427.92	19.30	133.85
Zn-0.3Li [140]	292	367.2	19.3	-
Zn-0.4Li [135,140]	352.2–363.7	398.5–406.5	27.4–27.8	-
Zn-0.8Li [141]	261.5	401.4	80.8	-
Zn-0.8Li-0.4Mn [142]	449.7	505.1	40.5	137.6
Zn-3Ag-0.5Mg [142]	385	432	34	-

**Table 11:** In vitro biocompatibility and hemocompatibility of Fe, Mg, Zn alloys used for stent applications.

Materials		Hemocompatibility	Reference
Fe	Pure Fe	~2.44%	[166]
	Fe-5wt.%Pd	Slightly higher than that of pure iron, but still less than 5%	[167]
	Fe-5wt.%Pt	Slightly higher than that of pure iron, but still less than 5%	[167]
	Fe30Mn	Below 2%	[110]
	Fe30Mn6Si	Below 2%	[110]
Zn	Pure Zn	1.04%	[34]
	Zn-0.8Cu	0.47%	[34]
	Zn-0.8Mn	0.57%	[34]
	Zn-0.8Li	0.52%	[34]
	Zn-3Cu	~1%	[168]
	Zn-Cu-Fe	~1%	[168]
	Zn-1.2Mg	1.62%	[132]
	Zn-1Mg-0.1Sr	1.10%	[169]
	Zn-1Mg-0.1Mn	1.10%	[170]
Mg	Pure Mg	Up to 37%	[171]
	Mg-OH	Below 5%	[162]
	Mg-HF	Below 5%	[162]
	Mg-P	Below 5%	[172]
	MG-PA	Below 5%	[173]
	Mg-1Zn-1Mn	65.75%	[173]
	Mg–Nd–Zn–Zr	52%	[162]
	Mg-Li	3~3.8%	[79,166]
	Mg8.5Li1Al	~4%	[174]
	Mg-Li-Al-RE	~4.2–7%	[174]

**Table 12:** In vivo experiments on Mg alloys.

Device	Backbone Material	Trial	Late Lumen Loss (mm)	Target Lesion Failure (%)	Reference
AMS	Magmaris	Follow up 63 patients for 12 months	1.08 ± 0.49 mm at 4 months	23.8% after 4 months, 45% after 1 year	[195]
DREAMS (Biotronik)	Magmaris, PLGA, Paclitaxe	Follow up 46 patients for 12 months	0.65 ± 0.50 mm at 4 months, 0.52 ± 0.39 mm at 12 months	4.3% at 12 months	[196]
		3-year follow-up	0.51 ± 0.46 mm at 12 months, 0.32 ± 0.32 mm at 28 ± 4 months	4.3% at 3 years	[197]
DREAMS 2G (Biotronik)	Magmaris, PLGA, Sirolimaus	Follow up 123 patients for 6 months	0.44 ± 0.36 mm at 6 months	1.7% at 6 months	[64]
		12-month follow-up	0.37 ± 0.25 mm at 6 months, 0.39 ± 0.27 mm at 12 months	1.7% at 12 months	[64]
		3-year follow-up	0.39 ± 0.27 mm at 12 months, 0.54 ± 0.38 mm at 36 months	4.3% at 36 months	[198]
Magmaris (Biotronik)	Magmaris, PLGA, Sirolimaus	Follow up 61 patients for 6 months	0.39 ± 0.39 mm	1.7% at 6 months	[198]
		Follow up 61 patients for 12 months	NA	1.6% at 12 months	[198]
		Follow up 1075 patients for 12 months	NA	3.9% at 12 months	[199]
		Follow up 2066 patients for 2 years	NA	6% at 24 months	[200]

**Table 13:** In vivo experiments on Fe alloys.

Device	Backbone Materials	Trial	Late Lumen Loss (mm)	Target Lesion Failure (%)	Reference
IBS (Lifetech)	Nitrided iron, Zn, PLA, Sirolimaus	Follow up 45 patients for 6 months	0.33 ± 0.27 mm	2.2% at 6 months	[66]
		Follow up 45 patients for 12 months	0.39 ± 0.50 mm	6.7% at 1 year	
		Follow up 45 patients for 24 months	0.40 ± 0.31 mm	6.7% at 2 years	
		Follow up 45 patients for 36 months	0.37 ± 0.57 mm	6.7% at 3 years	
NOR-I	>99.8% Pure Fe	Follow up 16 minipigs for 18 months	The loss of luminal area was less than 10%	-	[98]
Peripheral iron stent	>99.5% Fe	Follow up 29 patients for 360 days	There is no difference with the 316L stent. The loss of luminal area was less than 50%	-	[99]

**Table 14:** In vivo experiments on Zn alloys.

Animal Model	Material	Experiment Period	Biocompatibility	Degradation	Reference
Rat abdominal aorta	Pure zinc wire	6 months	None of the major contributors to restenosis observed	Retained about 70% area after 4 months, then degradation increased rapidly	[203]
	Pure zinc wire, Zn-1.5Mg wire, Zn-1.5Sr wire	1 month	No extensive inflammation was observed	Corrosion rate of Zn-based materials was 0.4 mm/year, significantly slower than that of AZ31	[204]
	Pure zinc stent	12 months	None of the major contributors to restenosis observed	Maintained mechanical integrity for 6 months and degraded 41.75 ± 29.72% of volume after 12 months	[205]
	Zn-5.5Al strip	6 months	Chronic and acute inflammatory indications were present	Cross-sectional reduction of pure Zn and Zn-Al alloys was 30–40% and 40–50% after 4.5 and 6 months	[176]
	Zn-0.1Li wire	12 months	Indicated moderate inflammation with a nonobstructive neointima	Indicated a moderate to low degradation rate of ~0.02 mm/year at 6.5 months, which increased to -0.05 mm/year at 12 months	[206]
Porcine coronary artery	Zn-0.8Cu stent	24 months	Endothelialization process could be completed in the first month. No inflammation responses or thrombosis formation were observed within 24 months	The implanted stent maintained its structural integrity after 6 months. After 24 months, approximately 28 ± 13 vol.% of the stent remained	[207]
Porcine iliofemoral artery	Zn-3Ag stent	6 months	Stent struts were completely covered by neointima at the 4-week follow-up; no stent thrombosis or vascular occlusion at 6 months	Maintained the stent for a minimum of 6 months	[208]
Rabbit carotid artery	Zn-0.02Mg-0.02Cu stent	12 months	Rapid endothelialization on the Zn-based alloy stent at 1 week, suggesting low cytotoxicity and thrombosis risk	Almost intact 6 months after implantation. Became incomplete, presented severe localized corrosion after 12 months	[209]

**Table 15:** Differences in the mechanical properties of different link types.

Reference	Objects	Method	Result
Behrend [223]	L-shaped, V-shaped, and S-shaped	Cantilever method	The L-shaped bridge had the smallest axial stiffness.
Ormiston et al. [224]	L-shaped, V-shaped, and S-shaped bridges	Three-point support method	The performance of the S-shaped vascular stent was better than that of the L-shaped and V-shaped stents, and the axial flexibility of the L-shaped and V-shaped stents was almost the same.
Wei et al. [225]	I-shaped, C-shaped, S-shaped, U-shaped, N-shaped, and W-shaped bridges	Finite element simulation of the ideal model analysis	When the vascular curvature was 0° or 15°, the stent with an S-shaped bridge structure was the most flexible. When the vascular curvature was 30°, 45°, or 60°, the U-shaped stent had the best flexibility.
Azaouzi et al. [219]	V-shaped, N-shaped, unsymmetrical V-shaped, and unsymmetrical N-shaped bridges	Finite element analysis	In terms of bending performance, the symmetrical N-shaped bridge and unsymmetrical V-shaped bridge had better flexibility; in terms of torsional performance, the symmetrical V-shaped bridge stent had the worst flexibility, and the unsymmetrical N-shaped stent had the best flexibility; since the radial force and stress of the symmetrical N-shaped bridge structure are small, it is the structure with the best radial support performance among the stents.
Wei et al. [226,227]	JS-shaped, OCS-shaped, and CCS-shaped bridges	Plane compression method, V-groove compression method, and three-point bending method	The radial strengths of the JS-shaped, open OCS-shaped, and closed CCS-shaped stents were 14%, 34%, and 42% higher than that of the ordinary biodegradable stent, respectively. The bending stiffness of the JS-shaped and OCS-shaped stents was similar to that of the ordinary stent, while the CCS-shaped stent had about 73% lower stiffness. All stents showed no axial foreshortening.
Mori and Saito [228]	W-shaped, S-shaped, WD-shaped, and N-shaped bridges	Four-point bending test method	The bending stiffness of the S-shaped stent was 85.28 N ${mm}^{2}$, that of the N-shaped stent was 41.67 N ${mm}^{2}$, that of the improved WD-shaped stent was 78.79 N ${mm}^{2}$, and that of the W-shaped stent was 188.67 N ${mm}^{2}$.

**Table 16:** Differences in the mechanical properties of different RVE stents.

Reference	Objects	Result
Prithipaul [216] and Douglas et al. [234]	Diamond, Re-entrant Auxetic, Hybrid A, Hybrid C, Chevron B stent	Diamond structure exhibited poor mechanical properties; Re-entrant Auxetic, Hybrid A, Hybrid C, and Chevron B exhibited better radial stiffness and foreshortening.
Dolla et al. [235] and Tan et al. [236]	Diamond, Auxetic stent	Re-entrant Auxetic stent had good mechanical properties.
Liu et al. [237]	Re-entrant shape memory polymer vascular stent	A re-entrant stent with a smaller radius has a higher critical buckling load and smaller buckling displacement. Compared to traditional stents, it has a smaller contact area with the vessel and lower stress after implantation.
Ruan [238]	Antichiral Reentrant vascular stent	Antichiral re-entrant stent had good mechanical properties after being implanted in the blocked lesion by designing stents of different sizes.

**Table 17:** The most significant advantages and concerns associated with the use of Mg-, Fe-, and Zn-based alloys in stents.

Alloy	Advantage	Concern
Mg-based	Excellent biocompatibility	High corrosion or biodegradation rate
Fe-based	Excellent mechanical properties; biological benefits: decreasing the proliferation of smooth muscle cells and inhibition of neointimal hyperplasia.	Low corrosion or biodegradation rate
Zn-based	Good biocompatibility; optimal degradation rate: 10 and 20 mm/year, similar to the ideal bioabsorbable material [155].	Cytotoxicity: excessive concentration of Zn ions can inhibit cell activity.

## Data Availability

No new data were created or analyzed in this study. Data sharing is not applicable to this article.

## Appendix A

**Table A1:** Test results for common Mg alloys used in biodegradable stents [70,256,257,258,259,260,261,262,263,264,265,266,267,268,269,270].

Material System	Stent Material	YS (Mpa)	UTS (Mpa)	EL (%)	Hardness	Degradation (mm·Year^−1^)		In Vitro Experiment Solution	Other Conditions
						Immersion Test	Potentiodynamic		
Mg Alloys	Pure Mg	83	143	9.8	-	-	-	-	-
	AZ31	172	-	16	-	1.50–1.92	-	Hanks’	5% CO_2_, 95% relative humidity, T = 37 °C
	AZ31B	198	230	12	-	-	-	-	-
	AZ91	98	172	4.3	-	-	-	-	-
	JDBM	123–220	267	26–48.8	-	-	-	-	-
	WE43	113	340–440	10.0–21	102–114	-	-	-	-
	ZE21B	196	298	20	-	-	2.16	-	-
	ZM21	340	353	11.5	-	-	-	-	-
	Mg-4Zn-1Y	240	330	20.4	-	-	-	-	-

R: ratio of surface area to solution volume in cm^2^/mL.

**Table A2:** Test results for common Fe alloys used in biodegradable stents [113,167,270,271,272,273,274,275].

Material System	Stent Material	YS (Mpa)	UTS (Mpa)	EL (%)	Hardness	Degradation (mm·Year^−1^)		In Vitro Experiment Solution	Other Conditions
						Immersion Test	Potentiodynamic		
Fe Alloys	Pure Fe	298.72–387.08	200–250	20–30	149.68–160.16	0.44–0.70 (static, day 30) 0.63–1.59 (dynamic, day 30)	0.043–0.071	Hanks’	37 ± 0.5 °C, pH = 7.4, DO (2.8–3.2 mg^−1^), shear stress (0.68 Pa)
	Nitrided Fe	585.32–643.38	300–350	10.0–15	278.39–295.73	-	0.223–0.227	-	-
	Fe-10Mn	650–800	1300–1400	14	367–434	3.97 (day 1), 2.05 (day 2)	-	SBF	T = 37 ± 1 °C
	Fe-10Mn-1Pd	850–950	1450–1550	2.0–11	370–440	6.68 (day 1), 9.05 (day 2)	-		
	Fe65Mn35	-	-	-	-	6.695 (day 30)	0.51	-	pH7.4 ± 0.2
	Fe65Mn35Ag1	-	-	-	-	19.01 (day 30)	0.96	-	
	Polished Fe–Mn	-	-	-	-	-	1.21	-	pH7.8
	Laser-textured Fe–Mn	-	-	-	-	-	8.63	-	-
	Fe–30Mn	-	-	-	-	1.08 (day 30)	0.127	PBS	T = 37 °C, pH = 7.4
	Fe–30Mn prepared by continuous laser	-	-	-	-	1.01 (day 30)	0.114		
	Fe–30Mn prepared by nanosecond laser	-	-	-	-	1.27 (day 30)	0.136		
	Fe–30Mn prepared by femtosecond laser	-	-	-	-	1.51 (day 30)	0.179		
	Fe–30Mn–Ag	-	-	-	-	-	0.012	Hanks’	-
	Fe-2.1Zn	235–267	402–440	18.3–23.3	134–136	4.56	0.238	SBF	T = 37 °C, pH = 7.4
	Fe-4.6Zn	208–284	437–489	12.6–20.8	146–150	5.77	0.323		
	Fe-7.2Zn	252–278	497–531	12.8–14.6	168–170	5.31	0.231		
	Fe-2Ag	210	-	-	-	0.72 (Static, day 30)	0.12	Hanks’	T = 37 ± 0.5 °C, pH = 7.4, DO (2.8–3.2 mg^−1^), dissolved oxygen, shear stress (0.68 Pa)
						1.74 (dynamic, day 30)	-		
	Fe-5Ag	380	-	-	-	0.90 (Static, day 30)	0.14		
						2.06 (dynamic, day 30)	-		
	Fe-10Ag	200	-	-	-	0.78 (Static, day 30)	0.175		
						1.87 (dynamic, day 30)	-		
	Fe-2Au	350	-	-	-	0.73 (Static, day 30)	0.174		
						1.73 (dynamic, day 30)	-		
	Fe-5Au	250	-	-	-	0.98 (Static, day 30)	0.131		
						2.31 (dynamic, day 30)	-		
	Fe-10Au	350	-	-	-	0.87 (Static, day 30)	0.098		
						1.72 (dynamic, day 30)	-		
	Fe-Pd	445	-	-	-	0.74 (Static, day 30)	-	Hanks’	T = 36.0–37.1 °C (immersion), T = 37 ± 0.5 °C (electrochemical), pH = 7.35 –7.45, DO (2.8–3.2 mg^−1^), shear stress (0.68 Pa)
	Fe-Pt	503	-	-	-	1.63 (dynamic, day 30)	-	-	-
	M-Fe	-	-	-	-	2.57	0.137	SBF	T = 37 °C, pH = 7.4
	E-Fe	270–360	292–423	-	-	3.48	0.139		
	Fe/0.3CNTs	-	-	-	-	2.05 (day 28)	0.090e	-	pH = 7.4–8, aerated
	Fe/0.6CNTs	-	-	-	-	2.27 (day 28)	0.160e	-	
	Fe/0.9CNTs	-	-	-	-	3.20 (day 28)	0.225e	-	
	Fe/1.2CNTs	-	-	-	-	3.30 (day 28)	0.241e	-	

R: ratio of surface area to solution volume in cm^2^/mL.

**Table A3:** Test results for common Zn alloys used in biodegradable stents [113,126,128,129,130,131,132,134,135,136,137,138,139,140,141,142,156,170,189,190,191,204,205,260,276,277,278,279,280,281,282,283,284,285,286,287,288,289,290].

Material System	Stent Material	YS (Mpa)	UTS (Mpa)	EL (%)	Hardness	Degradation (mm·Year^−1^)		In Vitro Experiment Solution	Other Conditions
						Immersion Test	Potentiodynamic		
Zn Alloys	Pure Zn	27.5–130	29.7–151	0.62–8.8	-	0.013–0.078 (static)	0.03–0.19	DPBS	pH = 7.4, agitation 80 rpm
								Ringer’	
								Human plasma	
								Whole blood	
	Ultra-pure Zn plate	-	-	-	-	0.013 (Static)	-	Hanks’ (30 days)	pH = 7.4, R = L125
	Ultra-pure Zn: laser cut tube	-	-	-	-	0.037 (Static)	-		
	Zn-0.08Mg	221	339	40	103	-	-	-	-
	Zn-0.15Mg	114	250	22	52	-	-	-	-
	Zn-0.5Mg	159–192	102.02–297	2.97–28	60.32–73	0.071 (0.0134 mg·cm·day) (Static)	-	DMEM, MEM (1 days)	T = 37 °C agitation 125 rpm
	Zn-0.8Mg	203	301	15	80–90	-	-	-	-
	Zn-1Mg	316–383	435–482	23–35	-	0.073–0.086 (Static)	0.149–0.169	aerated SBP (14 days)	T = 37 °C
	Zn-1.2Mg	219.61	362.64	21.31	96.01	-	-		
	Zn-1.5Mg	112.29–250	250.55–300	1.25–17.5	70–150	0.069 (Static)	-		
	Zn-3Mg	36–291	46–399	1–6.3	117–200	0.073 (Static)	-		
	Zn-4.2Mg	-	-	-	-	0.07–0.09 (Static)	0.12–0.19	Hanks’ (90 days)	T = 37 °C
	Zn-5Mg	-	-	-	-	0.101 (0.0 IP ms·cm·day) (Static)	-	DMEM, MEM (3 days)	T = 37 °C, pH = 7.4, 5% CO_2_: atmosphere
	Zn-5.0Mg-1.0Fe	-	-	-	-	0.062 (Static)	-	SBF (20 days)	T = 37 °C
	Zn-1Mg-0.1Ca	209.04	331.51	35.43	110–125	-	-	-	-
	Zn-1Mg-1Ca	-	-	-	-	0.090 (Static)	0.169	Hanks’ (30 days)	T = 37 °C
	Zn-1.0Mg-1.0Sr	-	-	-	-	0.095 (Static)	0.175	Hanks’ (30 days)	T = 37 °C
	Zn-1.0Mg-0.1Mn	114.10–195.02	131.94–299.04	1.11–26.07	97.66–107.82	0.051–0.070 (Static)	0.140–0.260	Hanks’ (48 days)	T = 37 °C, pH = 7.4, R = 1/25
	Zn-1.5Mg-0.1Mn	114.71	121.72	0.77	148.69	0.061 (Static)	0.25		
	Zn-1.5Mg-0.1Ca	173.81	241.72	1.72	150	0.115 (Static)	0.28	Hanks’ (20 days)	T = 37 °C, pH = 7.4, R = 1/25
	Zn-1.5Mg-0.1Sr	129.55	209.22	2.03	150	0.105 (Static)	0.106		
	Zn-5Mg-1Fe	176–187	223–231	22–26	-	0.040 (Static)	-	SBF (20 days)	T = 37 °C
	Zn-0.5Al	119	203	33	59	0.080 (Static)	-	SBF (30 days)	-
	Zn-0.5Al-0.1Mg	-	-	-	-	0.110 (Static)	-	SBF (30 days)	-
	Zn-0.5Al-0.3Mg	-	-	-	-	0.131 (Static)	-	-	-
	Zn-0.5Al-0.5Mg	-	92	1.73	94	0.150 (Static)	-	SBF (30 days)	T = 37 °C, pH = 7.6
	Zn-0.5Al-0.5Mg-0.1Bi	-	102	2.38	102	0.170 (Static)	-		
	Zn-0.5Al-0.5Mg-0.3Bi	-	108	2.74	109	0.210 (Static)	-		
	Zn-0.5Al-0.5Mg-0.5Bi	-	98	1.97	99	0.280 (Static)	-		
	Zn-1Al	134	223	24	73	-	-	-	-
	Zn-2Al-0.4Li	352.2	405.8	31.6	-	-	-	-	-
	Zn-4Al-0.2Li	362.7	382.2	33.3	-	-	-	-	-
	Zn-4Al-0.4Li	399.3	414.6	25.6	-	-	-	-	-
	Zn-4Al-0.6Li	429.6	451.4	46.3	-	-	-	-	-
	Zn-6Al-0.4Li	335.6	432.6	42.7	-	-	-	-	-
	Zn-4Al-lCu	-	-	-	-	0.075 (Static)	-	aerated SBP (14 days)	T = 37 °C, pH = 7
	Zn-2Al-2Cu-0.6Li	260	402	44	-	-	-	-	-
	Zn-2Al-2Cu-0.8Li	371	445	47	-	-	-	-	-
	Zn-2Al-4Cu-0.6Li	404	535	32	-	-	-	-	-
	Zn-2Al-4Cu-0.8Li	407	536	17	-	-	-	-	-
	Zn-1Cu	148.7	19.2–186.3	1.7–21	61.72	0.033 (Static)	–	SBF (20 days)	T = 37 °C, R = 1/25
	Zn-2Cu	199.7	68.5–240	2.77–46.8	68.78	0.026 (Static)	–		
	Zn-3Cu	128–213.7	105.5–288	4.06–47.2	67.04–76.64	0.012–0.030 (Static)	0.018		
	Zn-4Cu	227–250	270–270.7	50.6–51	-	0.025 (Static)	0.006		
	Zn-3Cu-0.1Mg	325–350	350–375	5.0–10.0	-	0.022 (Static)	0.024	Hanks’ (14 days)	T = 37 °C, R = 1/25
	Zn-3Cu-0.5Mg	400–425	400–425	0–5	-	0.030 (Static)	0.185		
	Zn-3Cu-1Mg	426.7	440.5	0.9	-	0.043 (Static)	0.104		
	Zn-3Cu-0.5Fe	269	284–302	24.7–32.7	76.06	0.064 (Static)	0.131	SBF	T = 37 °C
	Zn-3Cu-1Fe	-	272	19.6	82.24	0.069 (Static)	0.133		
	Zn-2.5Ag	147	203	32–36	-	0.079 (Static)	0.134	Modified Hanks’ renewed every two days (14 days)	T = 37 °C, pH = 7.4 5%, CO_2_ atmosphere, 90% humidity
	Zn-7Ag	236	287	32–36	-	0.084 (Static)	0.147		
	Zn-2Cu-0.3Li	226.55	333.49	26	124.59	-	-	-	-
	Zn-3Cu-0.5Fe	269	302	24.7	-	-	-	-	-
	Zn-3.5Cu-0.3Li	231.63	382.8	18.33	128.79	-	-	-	-
	Zn-4Cu-0.02Li	256	342	39.8	-	-	-	-	-
	Zn-5Cu-0.3Li	272.87	427.92	19.3	133.85	-	-	-	-
	Zn-0.3Li	292	367.2	19.3	-	-	-	-	-
	Zn-0.2Li	87.1	88.7	0.5	100	-	0.06	SBF	T = 37 °C, pH = 7.4, 5% CO_2_ atmosphere, R = 1/20
	Zn-0.4Li	352.2–363.7	398.5–406.5	27.4–27.8	-	-	0.05		
	Zn-0.8Li	261.5	401.4	80.8	-	-	-	-	-
	Zn-0.8Li-0.4Mn	449.7	505.1	40.5	137.6	-	-	-	-
	Zn-3Ag-0.5Mg	385	432	34	-	-	-	-	-
	Zn-1.0Ca	-	-	-	-	0.0591 (Static)	0.16	Hanks’ (14 days)	T = 37 °C
	Znl-1.0Sr	-	-	-	-	0.098 (Static)	0.176		
	Zn-1.0Ca-1.0Sr	-	-	-	-	0.095 (Static)	0.175	Hanks’ (30 days)	T = 37 °C

R: ratio of surface area to solution volume in cm^2^/mL.
